# CP5-Centered Parietal HD-tACS Is Associated with Improved Performance in a Smartphone-Based Shopping Task in Older Adults: A Behavioral and EEG Investigation

**DOI:** 10.3390/brainsci16070678

**Published:** 2026-06-27

**Authors:** Jiabao Hu, Yuhao Zhu, Mengdie Wang, Xiaorong Cheng, Xianfeng Ding, Zhao Fan

**Affiliations:** 1 Key Laboratory of Adolescent Cyberpsychology and Behavior (CCNU), Ministry of Education, Wuhan 430079, China; hjb1128@mails.ccnu.edu.cn (J.H.); md2023@mails.ccnu.edu.cn (M.W.); x.cheng@ccnu.edu.cn (X.C.); xianfengding@ccnu.edu.cn (X.D.); 2 School of Psychology, Central China Normal University, Wuhan 430079, China; 3 Key Laboratory of Human Development and Mental Health of Hubei Province, Wuhan 430079, China

**Keywords:** HD-tACS, older adults, working memory, EEG, smartphone-task performance

## Abstract

**Highlights:**

**What are the main findings?**

- In Experiment 1, 4-Hz CP5-centered parietal HD-tACS was associated with faster completion under high cognitive load, higher target selection accuracy, a reduced difficulty–time slope, and better 2-back performance in older adults.
- In the reduced-trial EEG extension (Experiment 2), active HD-tACS was associated with improved target selection accuracy; exploratory post-stimulation theta-power changes in posterior/parietal regions may have accompanied high-demand target-selection-accuracy improvement.

**What are the implications of the main findings?**

- CP5-centered parietal HD-tACS may support selected working-memory and attentional-control components of performance in a controlled smartphone-based shopping task.
- The exploratory EEG findings do not establish online entrainment or a definitive neural mechanism, but motivate larger, better-powered studies at the intersection of neuromodulation, cognitive aging and human–technology interaction.

**Abstract:**

Background/Objectives: Older adults often experience difficulties in smartphone use, especially when digital tasks require goal maintenance, visual search, sequential action, and response verification. Working memory and parietal theta-band activity may support these cognitively demanding operations, but it remains unclear whether a single session of theta-frequency high-definition transcranial alternating current stimulation (HD-tACS), centered over CP5 as a parietal scalp location intended to approximate the left inferior parietal region, is associated with short-term changes in smartphone-task performance in aging. Methods: This study examined performance in a controlled smartphone-based shopping task and exploratory post-stimulation EEG correlates. In Experiment 1, 40 older adults were randomly assigned to active HD-tACS or sham stimulation. In Experiment 2, 28 older adults completed a reduced-trial EEG extension of the same task with electroencephalography (EEG) recording before and after stimulation. Results: Active stimulation improved smartphone-task performance, including faster completion under high cognitive load, higher target selection accuracy, and reduced difficulty–time slope. Working-memory performance on a two-back task was also improved, and individual differences in working-memory gains were associated with improvements in smartphone-task efficiency. Active HD-tACS most strongly improved target selection accuracy, and exploratory post-stimulation theta-power changes in posterior/parietal regions may have accompanied high-demand target-selection-accuracy improvement. These neural findings should be interpreted cautiously because the omnibus EEG effects were trend-level, EEG–behavior correlations were based on a small active-stimulation subgroup, data-quality sensitivity analyses indicated artifact-related instability in theta-power estimates, and the full exploratory EEG–behavior correlation matrix did not survive FDR correction. Conclusions: These findings provide short-term behavioral evidence that CP5-centered parietal HD-tACS may support performance in a cognitively demanding smartphone-based task and motivate further work at the intersection of neuromodulation, cognitive aging, and human–technology interaction.

## 1. Introduction

Smartphones have become a central gateway through which people access information, communicate with others, manage services, engage in commerce, and participate in everyday social life [1]. For older adults, effective smartphone use can support autonomy, social connectedness, access to healthcare and public services, and overall quality of life. However, older adults remain disproportionately affected by the digital divide. Contemporary digital inequality is no longer limited to physical access to devices or the Internet; it increasingly concerns differences in digital skills, patterns of use, confidence, and the ability to benefit from technology in daily life. Studies of older adults have shown that Internet and technology use are shaped by age, education, income, prior experience, perceived relevance, technical interest, and social support, with digital exclusion being especially pronounced among the oldest-old groups [2,3,4,5,6,7]. Importantly, the relationship between age and technology adoption is also mediated by cognitive abilities, computer self-efficacy, and computer anxiety, suggesting that the digital divide in aging reflects not only social and infrastructural barriers but also cognitive and psychological constraints [2,4,5,8].

From a cognitive perspective, smartphone use is challenging because many everyday mobile tasks require users to coordinate perception, memory, attention, action, and learned interface knowledge across multiple steps. Older adults may need to maintain task goals, search visually dense interfaces, interpret icons and product labels, distinguish target information from distractors, update selections, monitor progress, detect errors, and confirm final responses. These demands are closely related to age-sensitive cognitive functions such as processing speed, working memory, attentional control, semantic processing, visuospatial attention, action selection, and executive monitoring. Cognitive function has also been associated with older adults’ use of digital devices in clinical aging contexts [9]. Human–computer interaction research has emphasized that age-related changes in cognition should be considered when designing and evaluating technology for older users [2,10]. Consistent with this view, studies of mobile-device usability have shown that older adults often experience difficulties when navigating diverse interface structures, hierarchical menus, changing interaction patterns, and unfamiliar application layouts [11,12]. Therefore, older adults’ smartphone difficulties should not be viewed merely as failures of motivation or familiarity. They can also be understood as difficulties in cognitively demanding human–smartphone interaction, especially when the task requires goal maintenance, semantic interpretation, visual search, sequential action, and response verification.

Recent work on technological cognition further suggests that smartphone interaction should be understood as a multidimensional form of human–technology engagement rather than as a single working-memory process. The integrated technological-cognition framework proposes that technology use depends on the interplay of causal or technical reasoning, semantic cognition, visuospatial skills, sensorimotor knowledge, and social learning within a distributed hub-and-processors architecture [13]. Smartphone use is closely aligned with this literature: users must interpret symbols and product categories, navigate spatially constrained interfaces, select actions according to learned interface rules, monitor outcomes, and often rely on socially acquired knowledge about how digital systems operate. Recent neurocognitive evidence also suggests that digital technologies may differ from mechanical technologies, with digital tools engaging relatively stronger conceptual, communicative, and social-cognitive components [14]. Thus, the present study does not assume that smartphone interaction can be reduced to working memory alone. Instead, it examines whether CP5-centered parietal stimulation may influence one demand-relevant working-memory/attentional-control component of performance within a broader technological-cognition context.

A further challenge is methodological. Much of the existing literature on older adults’ technology use relies on questionnaires, interviews, or broad adoption measures, which are valuable for identifying attitudes, barriers, and usage patterns but do not directly capture moment-to-moment task performance. Conversely, traditional laboratory cognitive tasks offer strong experimental control but often lack the ecological structure of real-world smartphone interaction. This gap is important because everyday digital behavior is usually sequential, visually guided, and goal-directed rather than a single isolated cognitive response. Ecologically grounded performance tasks are therefore needed to connect cognitive mechanisms with real-world technology use. Smartphone shopping provides a suitable context for this purpose because it is familiar to many users while also requiring core operations common to broader smartphone interaction, including maintaining goals, searching for relevant information, selecting items, checking intermediate outcomes, and confirming a final response. Previous studies have examined mobile shopping in older adults as an important form of digital participation, showing that its adoption and use are influenced by factors such as perceived usefulness, effort expectancy, facilitating conditions, habit, social influence, and support from close others [15,16]. Research on mobile commerce further indicates that shopping through smartphone interfaces requires users to search for information, compare options, evaluate products, and complete transaction-related decisions within constrained screen environments [17,18]. Building on this literature, the present study used smartphone shopping as an ecologically familiar but experimentally controllable context for modeling cognitively demanding smartphone-task performance, rather than as a measure of consumer behavior per se.

Working memory is a likely cognitive mechanism underlying performance in such tasks. Working memory supports the temporary maintenance and manipulation of information, the updating of task-relevant representations, and the coordination of ongoing behavior [19,20]. During a complex smartphone task, users must maintain the current goal, remember which items or actions remain relevant, update the status of completed selections, and monitor whether the final response matches the intended goal. As task difficulty increases, these demands become stronger. The N-back task is widely used to assess working-memory updating and monitoring, and neuroimaging meta-analyses have consistently shown that N-back performance engages a distributed frontoparietal network [21,22]. In aging, working memory is often vulnerable, but performance varies substantially across individuals depending on neural maintenance, reserve, compensation, and task demands [23,24]. These findings suggest that interventions targeting working-memory-related neural systems may have the potential to improve not only laboratory working-memory performance but also complex real-world digital behaviors.

A CP5-centered parietal target was selected because parietal regions are involved in visual working memory, attentional allocation, goal maintenance, and the control of behavior under cognitive demand. Visual short-term memory research has shown that the posterior parietal cortex is closely related to capacity-limited visual representations [25]. Broader meta-analytic evidence also implicates parietal and frontoparietal regions in working-memory tasks across different domains and task requirements [21,22]. Recent technological-cognition work further highlights the left inferior parietal region, including area PF within the supramarginal gyrus, as a technical hub within broader fronto-temporo-parietal networks supporting interaction with human-made technologies [26,27]. At the same time, smartphone use is not a purely parietal or technical-reasoning process; it also recruits semantic, visuospatial, executive, and social-cognitive systems. Accordingly, the present CP5-centered montage was intended to approximate a demand-relevant left parietal region, but should not be interpreted as anatomically precise individualized IPL stimulation because MRI-guided neuronavigation and participant-specific current-flow modeling were not performed.

Transcranial alternating current stimulation (tACS) provides a non-invasive method for modulating rhythmic neural activity and testing causal links between oscillatory dynamics and cognition. Reviews of tACS indicate that rhythmic electrical stimulation can interact with ongoing brain oscillations and influence perception, motor function, and higher cognitive processes [28,29,30]. Theta-band oscillations have been closely linked to memory, cognitive control, working memory, and task monitoring [31,32,33,34]. In working-memory contexts, theta activity is thought to support the coordination of distributed neural activity and the control of task-relevant information [35]. Stimulation studies have provided converging evidence that modulating rhythmic activity can influence working-memory performance. For example, externally induced frontoparietal synchronization has been shown to modulate network dynamics and enhance working memory [36]. In older adults, rhythmic stimulation has been reported to improve working-memory function and alter age-related neural dynamics [37], while repeated stimulation protocols can produce dissociable improvements in working memory and long-term memory [38]. EEG-informed theta-frequency tACS has also been associated with memory-capacity changes and oscillatory aftereffects [39]. Together, this literature provides a strong rationale for targeting theta-related activity within working-memory networks.

High-definition stimulation may be especially suitable when a relatively constrained scalp montage is desired. A 4 × 1 high-definition montage uses a central electrode surrounded by return electrodes, allowing current to be concentrated more locally around the target region than conventional large-pad montages. Although high-definition montage work has often been described in the context of HD-tDCS, the same spatial logic is relevant for HD-tACS protocols that aim to modulate rhythmic activity within a more constrained cortical region [40]. Applying CP5-centered parietal theta-frequency HD-tACS therefore offers a theoretically grounded approach to modulating one parietal component of a broader working-memory and attentional-control network. If such stimulation is associated with improved older adults’ smartphone-task performance, it would suggest that neuromodulation of task-relevant cognitive-control systems may influence selected components of performance in a controlled smartphone-based task.

However, an important translational gap remains. Most studies of tACS and cognitive aging have focused on laboratory tasks such as working-memory span, delayed recall, or perceptual decision-making. These tasks are valuable for isolating cognitive processes, but they do not directly test whether stimulation-induced cognitive benefits extend to everyday technology-related behavior. Smartphone interaction is multidimensional: performance may improve through faster completion, more accurate target selection, reduced sensitivity to task difficulty, or better integrated speed–accuracy control. These outcomes may be related but are not identical. A stimulation protocol that improves working memory may therefore influence some aspects of smartphone-task performance more strongly than others. To evaluate ecological transfer, it is necessary to combine controlled cognitive measures with smartphone-based task performance that captures goal maintenance, visual search, sequential action, and response verification.

It is also important to examine whether behavioral effects are accompanied by neural changes that are consistent with the proposed mechanism. EEG provides a way to assess task-related oscillatory activity during smartphone-task performance. In the context of theta-frequency HD-tACS, a theoretically meaningful pattern would be that behavioral improvement is accompanied by changes in theta-band power, particularly within posterior or parietal regions related to the stimulation target and task demands. At the same time, mechanistic claims should be made cautiously. When EEG is recorded before and after stimulation, rather than during stimulation, the data can support conclusions about post-stimulation task-related oscillatory modulation, but they cannot by themselves prove online entrainment [41]. Therefore, EEG evidence in this context should be interpreted as preliminary neural evidence that may accompany behavioral improvement, rather than as definitive proof of direct stimulation-period entrainment.

The present study examined whether a single session of 4 Hz CP5-centered parietal HD-tACS was associated with short-term changes in older adults’ performance in a controlled smartphone-based shopping task and whether behavioral changes were accompanied by exploratory post-stimulation EEG correlates. The task was designed to model selected smartphone-shopping operations, including goal maintenance, visual search, target selection, cart checking, and final confirmation, while task difficulty was experimentally manipulated by varying the number of target products. Smartphone-task performance was assessed through completion time, target selection accuracy, and difficulty–time slope, and working memory was assessed with a two-back task.

We conducted two experiments with complementary purposes. Experiment 1 served as the main behavioral test of whether active CP5-centered parietal HD-tACS, relative to sham stimulation, was associated with changes in smartphone-task performance and two-back working-memory performance. We expected the clearest behavioral differences under higher task demands. Experiment 2 incorporated EEG during pre- and post-stimulation smartphone-task performance. Because the number of trials was reduced to accommodate EEG recording and reduce participant burden, Experiment 2 was designed as a reduced-trial EEG extension rather than a fully powered behavioral replication of all Experiment 1 effects.

Together, this two-experiment design examined a cautiously framed account of smartphone-task performance in aging. Experiment 1 provided the main short-term behavioral test, whereas Experiment 2 examined whether behavioral changes were accompanied by exploratory post-stimulation EEG correlates. The study was not designed to establish anatomically precise IPL targeting, online entrainment, causal mediation by theta activity, or general real-world smartphone competence.

## 2. Experiment 1: Effects of CP5-Centered Parietal HD-tACS on Older Adults’ Smartphone-Task Performance and Working Memory

Experiment 1 examined whether a single session of theta-frequency CP5-centered parietal high-definition transcranial alternating current stimulation (HD-tACS) was associated with changes in older adults’ performance in a controlled smartphone-based shopping task and in two-back working-memory performance. The smartphone task was implemented as a shopping paradigm because it involved selected components of human–smartphone interaction, including goal maintenance, visual search, item selection, cart checking, and final response confirmation. We hypothesized that, relative to sham stimulation, active HD-tACS would be associated with improved performance, particularly under higher task demands, and with changes in two-back working-memory performance.

### 2.1. Materials and Methods

#### 2.1.1. Participants

Forty older adults aged 60 years or above [42] were recruited from the local community. (Sample size was determined a priori using G*Power 3.1 [43,44]. For a 2 × 2 × 3 mixed-design ANOVA with stimulation group as the between-subjects factor and measurement time and task difficulty as within-subject factors, a minimum of 24 participants was required to detect a medium effect size, f = 0.25, with α = 0.05 and power = 0.90. Recruitment continued until 40 participants had been enrolled.) All participants were right-handed, had normal or corrected-to-normal vision, reported no hearing impairment, and had daily experience using smartphones. Participants were screened for basic eligibility, including self-reported physical and mental health status, recent major surgery, visual and hearing status, and handedness. Before the experiment, all participants underwent safety screening for transcranial electrical stimulation. Participants were excluded if they had a history of epilepsy or seizure-like episodes, metal implants in the head, implanted electronic devices, or current use of psychoactive medication. The final sample included 40 participants aged 60–74 years (*M* = 65.18, *SD* = 4.11). Participants were randomly assigned to either the active HD-tACS group (*n* = 20; 6 men, 14 women) or the sham stimulation group (*n* = 20; 5 men, 15 women). The two groups did not differ significantly in age, *t*(38) = −0.343, *p* = 0.734, or sex distribution, χ^2^(1) = 0.125, *p* = 0.723.

#### 2.1.2. Controlled Smartphone-Based Shopping Paradigm

Smartphone-task performance was assessed using a custom-developed Android application. The task was designed as an ecologically oriented but experimentally controlled smartphone-based shopping paradigm that preserved selected operations of everyday smartphone interaction while maintaining experimental control over task difficulty and stimulus presentation. Specifically, the task simulated product search, target selection, cart checking, and final submission. It was not intended to examine consumer behavior per se; rather, it served as an experimentally controlled human–smartphone interaction paradigm involving goal maintenance, visual search, sequential action, and response verification within a smartphone-like context under experimentally manipulated cognitive load.

In each trial, participants viewed a vertically scrollable interface containing 32 photographs of common consumer products, each accompanied by a text label. Product items were selected from four categories: vegetables, fruits, meat, and seafood. Product positions were randomized across trials.

**Figure 1:**
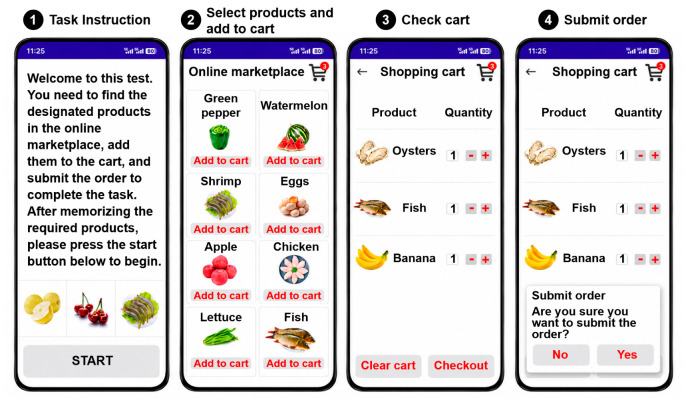
Example workflow of the controlled smartphone-based shopping task. The numbered steps illustrate task instruction, product search and target selection, cart checking, and order submission. Participants completed each trial by selecting the instructed products, verifying the cart contents, and confirming the order. The task interface was presented in Chinese during testing; English annotations are provided for clarity.

At the beginning of each trial, participants were instructed to find a set of target products. Task difficulty was manipulated by varying the number of target products: three targets in the low-difficulty condition, four targets in the medium-difficulty condition, and five targets in the high-difficulty condition. Participants searched the product list, tapped the “Add to cart” button below each target product, entered the cart page by tapping the shopping-cart icon, checked the selected items, and finally tapped the “Submit” button to complete the trial. To submit a trial, participants were required to retain exactly the same number of products in the cart as the number of instructed target products; the application did not allow submission when the number of retained products was lower or higher than the required target count. Participants could switch between the product page and cart page without restriction before submission, as illustrated in Figure 1.

The smartphone-task paradigm included three primary dependent variables. First, task completion time was defined as the time from trial onset to final submission. Second, target selection accuracy was defined as the proportion of instructed target products correctly retained in the submitted response. Let *K* denote the number of instructed target products in a trial and *TP* denote the number of correctly retained target products. Because participants were required to submit exactly *K* products, each distractor product retained in the submitted response necessarily displaced one instructed target product. Thus, target selection accuracy was calculated as *TP*/*K*, and the final distractor selection rate was calculated as (*K* − *TP*)/*K*, which was equal to 1 − target selection accuracy. Target hits were defined as correctly retained target products, and target omissions were defined as instructed target products not retained in the submitted response. Under this fixed-cardinality rule, complete-order success was defined as trials in which all instructed target products were correctly retained in the submitted response, corresponding to target selection accuracy of 100%. Third, the difficulty–time slope was calculated for each participant by regressing mean completion time on task difficulty coded as the number of target products. A smaller slope indicated that completion time was less affected by increasing task demands.

#### 2.1.3. Working-Memory Task

**Figure 2:**
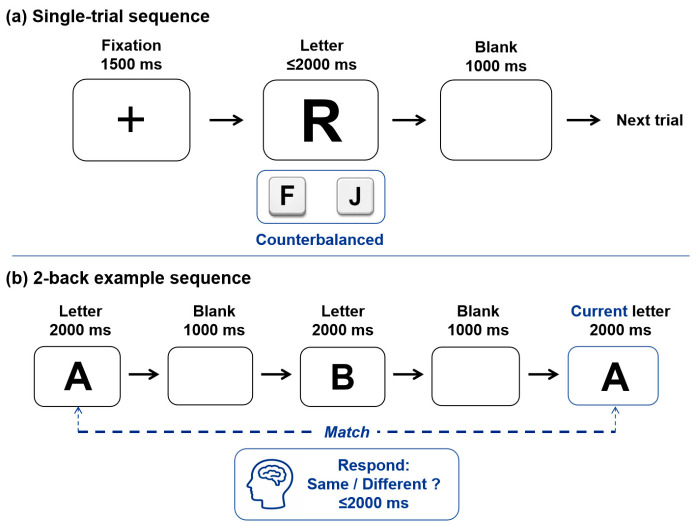
Structure and example sequence of the two-back working-memory task. (**a**) The single-trial sequence consisted of a fixation cross (1500 ms), letter presentation and response window (up to 2000 ms), and blank interval (1000 ms). Response-key mapping (F/J) was counterbalanced across participants. (**b**) Example sequence of the two-back task; the dashed line links the current letter to the matching letter presented two trials earlier.

Working memory was assessed using a two-back task programmed in PsychoPy (version 2023.1.3; Open Science Tools Ltd., Nottingham, UK), following the widely used N-back working-memory paradigm [21] (see Figure 2). The stimuli were 26 uppercase English letters. At the beginning of each trial, a fixation cross was presented for 1500 ms, followed by a letter stimulus. Participants judged whether the current letter matched the one presented two trials earlier. Participants responded by pressing one of two keys, F or J, with the mapping between response type and response key counterbalanced across participants. Each letter remained on the screen until a response was made or for a maximum of 2000 ms, followed by a 1000 ms blank interval. The task included 40 trials.

Three dependent variables were calculated for the two-back task: reaction time (*RT*), accuracy (*AC*), and the Linear Integrated Speed–Accuracy Score (*LISAS*). *RT* was calculated from correct-response trials. *AC* was defined as the proportion of correct responses. *LISAS* was used to integrate speed and accuracy into a single performance index, with lower values indicating better combined performance. *LISAS* was selected because it adjusts *RT* by error proportion while preserving the response-time metric and is less vulnerable than inverse efficiency scores to nonlinear inflation when error rates differ across conditions. Diffusion-model parameters were not estimated because the two-back task contained a limited number of trials, and the smartphone-shopping task did not consist of homogeneous binary decision trials. Following [45], *LISAS* was computed as

$$LISAS=RT+PE\times \left(\frac{{SD}_{RT}}{{SD}_{PE}}\right),$$

where *RT* is the mean reaction time, *PE* is the proportion of errors, *SD_RT_* is the standard deviation of reaction times, and SD*_PE_* is the standard deviation of error proportions. This score adjusts reaction time by incorporating error proportion and is therefore useful for summarizing performance when speed–accuracy trade-offs may be present.

#### 2.1.4. Apparatus and HD-tACS Protocol

The smartphone shopping paradigm was administered on a OnePlus 8 smartphone (OnePlus Science and Technology Co., Ltd., Shenzhen, Guangdong, China) running Android 12.0, with 12 GB RAM, a 6.55-inch display, a refresh rate of 90 Hz, a screen resolution of 2400 × 1080 pixels, and a pixel density of 402 ppi. The two-back task was presented on an iiyama HM903DT CRT monitor (iiyama Corporation, Tokyo, Japan), configured at a refresh rate of 60 Hz and a resolution of 1280 × 1024 pixels.

**Figure 3:**
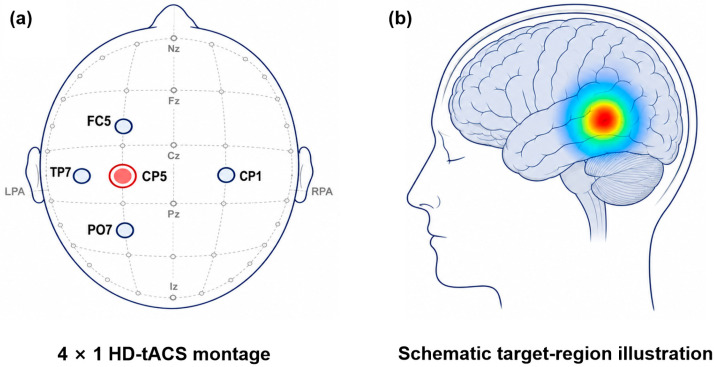
CP5-centered 4 × 1 HD-tACS montage and conceptual target-region illustration. (**a**) CP5 served as the central stimulation electrode (red), with TP7, FC5, CP1, and PO7 as surrounding return electrodes (blue). (**b**) Conceptual illustration of the intended parietal target region around the CP5-centered montage. Warmer colors indicate the conceptualized central target area, whereas cooler colors indicate more peripheral areas. This panel is not a computational electric-field simulation and does not represent participant-specific current-flow modeling.

HD-tACS was delivered using an LTE-tDCS stimulator with a 4 × 1 HD adaptor (Soterix Medical, Inc., Woodbridge, NJ, USA). Active stimulation was delivered at 4 Hz for 20 min with a peak current intensity of 1.5 mA. Electrode locations were identified using a cap compatible with the international 10–10 system. The 4 Hz stimulation frequency was selected to target theta-band activity, given prior evidence linking theta-band synchronization and theta-frequency tACS to working-memory and memory-related processes [36,37,39]. Circular Ag/AgCl electrodes with a diameter of 1 cm were positioned at CP5, TP7, FC5, CP1, and PO7. CP5 served as the central electrode, and TP7, FC5, CP1, and PO7 served as surrounding return electrodes, each receiving 25% of the return current (see Figure 3). We describe this montage as CP5-centered parietal HD-tACS because scalp-based CP5 placement was intended to approximate the left inferior parietal region but did not provide individualized anatomical targeting. Individualized MRI-guided neuronavigation or participant-specific current-flow modeling was not performed; therefore, anatomical specificity should be interpreted with caution.

In the sham condition, stimulation ramped up briefly at the beginning and end of the stimulation period and then returned to zero, thereby mimicking the initial sensation of stimulation without delivering sustained alternating current.

During HD-tACS, electrode impedance was continuously monitored and kept below 2 kΩ. Participants were monitored for discomfort and tolerance throughout stimulation. After the experiment, participants were assessed for possible adverse effects, including scalp tingling, itching, burning sensations, headache, and dizziness. No participant reported notable adverse effects. At debriefing, participants were informally asked whether they had noticed any clear differences or cues that allowed them to identify the stimulation condition. No participant reported being able to clearly distinguish between the active and sham conditions. Because forced-choice condition guesses, confidence ratings, and systematic adverse-sensation ratings by group were not collected, this procedure should be interpreted as an informal debriefing check rather than as a formal quantitative blinding assessment.

#### 2.1.5. Procedure

Each participant completed the experiment across two sessions conducted on separate days. On the first day, demographic information was collected, and participants were introduced to the experimental equipment, task requirements, and procedures. They then completed practice trials for both the smartphone shopping paradigm and the two-back task until they understood the task instructions. In the formal pre-stimulation session, participants first completed the smartphone task and then the two-back task.

The smartphone task consisted of three blocks corresponding to the low-, medium-, and high-difficulty conditions. Each block included 20 analyzable trials, yielding 60 trials in total. Block order was counterbalanced across participants. After completing the smartphone task, participants performed the two-back task.

**Figure 4:**
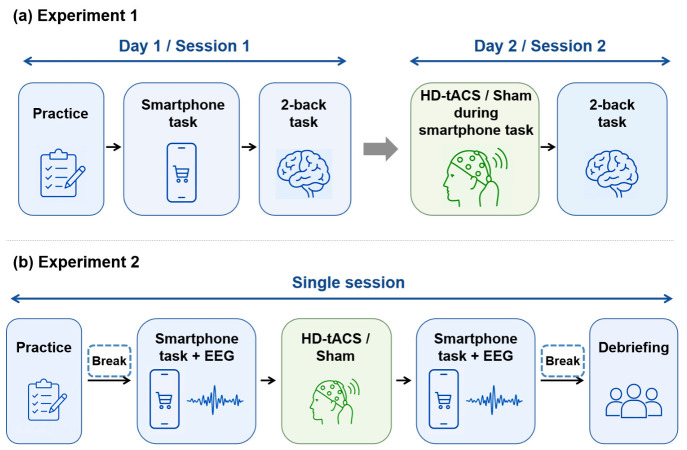
Experimental timelines for Experiments 1 and 2. (**a**) Experiment 1 was completed across two days: practice, smartphone-task performance, and two-back performance on Day 1; active HD-tACS or sham stimulation during the smartphone task and a subsequent two-back task on Day 2. (**b**) Experiment 2 was completed in a single session and included practice, pre-stimulation smartphone-task performance with EEG recording, CP5-centered parietal HD-tACS or sham stimulation, and post-stimulation smartphone-task performance with EEG recording. Arrows indicate task order; breaks are shown where applicable.

On the second day, participants received 20 min of active HD-tACS or sham stimulation while performing the smartphone shopping paradigm using a different set of product stimuli. Immediately after stimulation, participants completed the two-back task again. The procedure of Experiment 1 is shown in Figure 4a.

#### 2.1.6. Statistical Analysis

Baseline group differences in age, sex distribution, pre-stimulation smartphone-task performance, and pre-stimulation two-back performance were examined before testing stimulation effects. Because the study was not preregistered, this inferential hierarchy was defined retrospectively and should be interpreted with caution. To improve transparency, high-difficulty completion time was treated as the primary behavioral endpoint in Experiment 1 because the theoretical rationale predicted stronger stimulation-related benefits under higher cognitive demand. Target selection accuracy and difficulty–time slope were treated as key secondary smartphone-task outcomes; two-back accuracy and *LISAS* were treated as secondary cognitive outcomes, whereas working-memory correlations were treated as exploratory and hypothesis-generating. Task completion time and target selection accuracy in the smartphone paradigm were analyzed using 2 × 2 × 3 mixed-design analyses of variance (ANOVAs), with stimulation group (active HD-tACS vs. sham stimulation) as the between-subjects factor and measurement session (pre-stimulation vs. post-stimulation) and task difficulty (low, medium, or high) as within-subject factors. The difficulty–time slope was analyzed using a 2 × 2 mixed-design ANOVA, with stimulation group as the between-subjects factor and measurement time as the within-subject factor.

To address trial-level variability, we additionally conducted trial-level sensitivity analyses for Experiment 1 using generalized estimating equations with participant-level clustering [46]. Completion time was modeled at the trial level after log transformation, with stimulation group, measurement session, task difficulty, and their interactions as predictors. The trial-level analyses were treated as sensitivity analyses supporting the aggregated ANOVA results. Principal change-score effects are reported with 95% confidence intervals where appropriate. FDR correction families were specified separately for behavioral correlations, EEG frequency-band/ROI analyses, follow-up tests, and EEG–behavior correlations.

For the two-back task, *RT*, *AC*, and *LISAS* were analyzed separately using 2 × 2 mixed-design ANOVAs with stimulation group as the between-subjects factor and measurement time as the within-subject factor. Follow-up tests were conducted when theoretically relevant interaction effects were significant. Bonferroni correction was applied to post hoc comparisons involving multiple task-difficulty levels. Greenhouse–Geisser correction was applied where the sphericity assumption was violated. Statistical analyses were conducted using IBM SPSS Statistics (version 27.0.1; IBM Corp., Armonk, NY, USA).

To examine whether stimulation-related improvements in working memory were associated with improvements in smartphone-task performance, improvement scores were calculated for the active HD-tACS group. For completion time and difficulty–time slope, improvement was coded as pre-stimulation minus post-stimulation, such that larger positive values reflected greater improvement. For target selection accuracy and two-back accuracy, improvement was coded as post-stimulation minus pre-stimulation. For *LISAS*, improvement was coded as pre-stimulation minus post-stimulation. Pearson correlations were calculated between two-back improvement indices and smartphone-task improvement indices. To reduce the risk of false-positive findings, the Benjamini–Hochberg false discovery rate (FDR) correction was applied across the correlation family [47].

### 2.2. Results

Descriptive statistics for smartphone-task performance and two-back working-memory performance are reported in Table 1 and Table 2, respectively. Values are reported as mean (standard deviation). Target selection accuracy was defined as the proportion of instructed target products correctly retained in the submitted response. Lower completion time, lower difficulty–time slope, and lower *LISAS* indicate better performance, whereas higher target selection accuracy indicates better performance.

Full ANOVA summaries, baseline checks, and follow-up analyses are provided in Supplementary Tables S3, S5 and S6.

#### 2.2.1. Baseline Comparisons

At baseline, the active HD-tACS and sham stimulation groups did not differ significantly in smartphone-task completion time, target selection accuracy, or difficulty–time slope (all *ps* > 0.34). The two groups also did not differ significantly in pre-stimulation two-back accuracy or *LISAS* (both *ps* > 0.29). The baseline group difference in two-back *RT* was not significant (*t*(38) = −1.720, *p* = 0.094). Therefore, subsequent analyses focused on Group × Time interactions and within-participant pre-to-post changes.

#### 2.2.2. Effects of HD-tACS on Smartphone-Task Performance

##### Completion Time

For smartphone-task completion time, the main effect of task difficulty was significant (*F*(2, 76) = 139.760, *p* < 0.001, η^2^_p_ = 0.786), indicating that participants required more time as the number of target products increased. The main effect of measurement time was also significant (*F*(1, 38) = 9.314, *p* = 0.004, η^2^_p_ = 0.197), reflecting an overall reduction in completion time from pre- to post-stimulation. The main effect of the stimulation group was not significant (*F*(1, 38) = 0.165, *p* = 0.687, η^2^_p_ = 0.004).

Importantly, the Group × Time interaction was significant (*F*(1, 38) = 4.447, *p* = 0.042, η^2^_p_ = 0.105), and this interaction was further qualified by a significant Group × Time × Difficulty interaction (*F*(2, 76) = 4.756, *p* = 0.011, η^2^_p_ = 0.111). Follow-up comparisons showed that the active HD-tACS group exhibited a significant pre-to-post reduction in completion time specifically under the high-difficulty condition (*t*(19) = −4.671, Bonferroni-corrected *p* < 0.001, *d*z = −1.044). No significant pre-to-post decrease was observed in the low- or medium-difficulty conditions after correction. In the sham stimulation group, completion time did not significantly change after stimulation at any difficulty level (all corrected *ps* > 0.99).

Trial-level sensitivity analyses supported the aggregated ANOVA pattern. A Gaussian GEE model for log-transformed completion time showed a significant Group × Time × Difficulty effect, χ^2^(2) = 13.39, *p* = 0.001. A focused high-difficulty model also showed a significant Group × Time effect, χ^2^(1) = 15.19, *p* < 0.001, indicating that the high-demand completion-time effect was not dependent on aggregation across trials (Supplementary Table S7).

##### Target Selection Accuracy

For target selection accuracy, the main effect of task difficulty was significant (*F*(2, 76) = 138.259, *p* < 0.001, η^2^_p_ = 0.784), indicating that participants selected a smaller proportion of target items as task difficulty increased. The main effect of measurement time was significant (*F*(1, 38) = 47.334, *p* < 0.001, η^2^_p_ = 0.555), reflecting an overall increase in target selection accuracy after stimulation. The main effect of the stimulation group was not significant (*F*(1, 38) = 0.263, *p* = 0.611, η^2^_p_ = 0.007).

Critically, the Group × Time interaction was significant (*F*(1, 38) = 24.537, *p* < 0.001, η^2^_p_ = 0.392). The Time × Difficulty interaction was also significant (*F*(2, 76) = 4.006, *p* = 0.022, η^2^_p_ = 0.095). The Group × Difficulty interaction and the Group × Time × Difficulty interaction were not significant (both *ps* > 0.10). Follow-up comparisons showed that the active HD-tACS group exhibited significant increases in target selection accuracy after stimulation in the low-difficulty condition (*t*(19) = 2.866, Bonferroni-corrected *p* = 0.030, *d*z = 0.641); medium-difficulty condition (*t*(19) = 2.926, Bonferroni-corrected *p* = 0.026, *d*z = 0.654); and high-difficulty condition (*t*(19) = 6.538, Bonferroni-corrected *p* < 0.001, *d*z = 1.462). In contrast, no significant pre-to-post changes were observed in the sham stimulation group at any difficulty level (all corrected *ps* > 0.50). Descriptive statistics for target hits, target omissions, target selection accuracy, the derived final distractor selection rate, and complete-order success are provided in Supplementary Table S8. Under the fixed-cardinality submission rule, the final distractor selection rate was equal to 1 − target selection accuracy and was therefore not analyzed as an independent outcome.

##### Difficulty–Time Slope

For the difficulty–time slope, the main effect of measurement time was significant (*F*(1, 38) = 19.039, *p* < 0.001, η^2^_p_ = 0.334), indicating that completion time became less strongly affected by increasing task difficulty after stimulation. The main effect of the stimulation group was not significant (*F*(1, 38) = 0.501, *p* = 0.484, η^2^_p_ = 0.013). The Group × Time interaction was significant (*F*(1, 38) = 14.210, *p* < 0.001, η^2^_p_ = 0.272). Follow-up comparisons showed that the active HD-tACS group exhibited a significant decrease in difficulty–time slope after stimulation (*t*(19) = −4.335, *p* < 0.001, *d*z = −0.969), whereas the sham stimulation group showed no significant change (*t*(19) = −0.857, *p* = 0.402, *d*z = −0.192).

**Figure 5:**
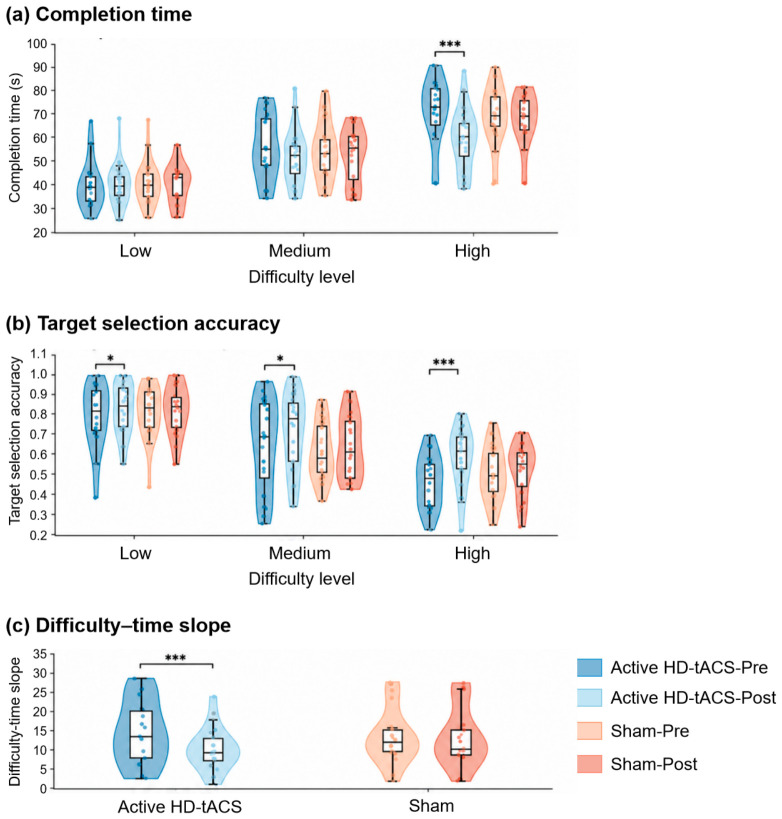
Smartphone-task performance in Experiment 1. (**a**) Completion time across low-, medium, and high-difficulty conditions. (**b**) Target selection accuracy across difficulty conditions. (**c**) Difficulty–time slope. Colored dots represent individual participants; violin width represents the distribution density; and white boxplots show the median (central line) and interquartile range. Colors distinguish the active HD-tACS and sham groups at pre- and post-stimulation. Brackets and asterisks indicate significant Bonferroni-adjusted within-group pre–post comparisons. * *p* < 0.05, *** *p* < 0.001.

Together, these findings indicate that active HD-tACS improved older adults’ performance in the controlled smartphone-based shopping task, as reflected by faster completion under high task demands, higher target selection accuracy, and reduced susceptibility of completion time to increasing task difficulty (Figure 5).

#### 2.2.3. Effects of HD-tACS on Two-Back Working-Memory Performance

For two-back *RT*, the main effect of stimulation group was significant (*F*(1, 38) = 7.813, *p* = 0.008, η^2^_p_ = 0.171), indicating overall faster responses in the active HD-tACS group than in the sham stimulation group. The main effect of measurement time was not significant (*F*(1, 38) = 0.767, *p* = 0.387, η^2^_p_ = 0.020). The Group × Time interaction was marginal but did not reach significance (*F*(1, 38) = 3.595, *p* = 0.066, η^2^_p_ = 0.086).

For two-back accuracy, the main effect of measurement time was significant (*F*(1, 38) = 78.932, *p* < 0.001, η^2^_p_ = 0.675), and the main effect of stimulation group was also significant (*F*(1, 38) = 5.561, *p* = 0.024, η^2^_p_ = 0.128). More importantly, the Group × Time interaction was significant (*F*(1, 38) = 63.410, *p* < 0.001, η^2^_p_ = 0.625). Follow-up comparisons showed that accuracy increased substantially after stimulation in the active HD-tACS group (*t*(19) = 9.660, *p* < 0.001, *d*z = 2.160), whereas no significant change was observed in the sham stimulation group (*t*(19) = 0.941, *p* = 0.358, *d*z = 0.210).

For *LISAS*, the main effect of stimulation group was significant (*F*(1, 38) = 10.291, *p* = 0.003, η^2^_p_ = 0.213), whereas the main effect of measurement time was not significant (*F*(1, 38) = 0.324, *p* = 0.572, η^2^_p_ = 0.008). The Group × Time interaction was significant (*F*(1, 38) = 12.892, *p* < 0.001, η^2^_p_ = 0.253). Follow-up comparisons showed that *LISAS* decreased significantly after stimulation in the active HD-tACS group (*t*(19) = −3.787, *p* = 0.001, *d*z = −0.847), indicating improved integrated speed–accuracy performance. The sham stimulation group did not show a significant change (*t*(19) = 1.808, *p* = 0.087, *d*z = 0.404).

**Figure 6:**
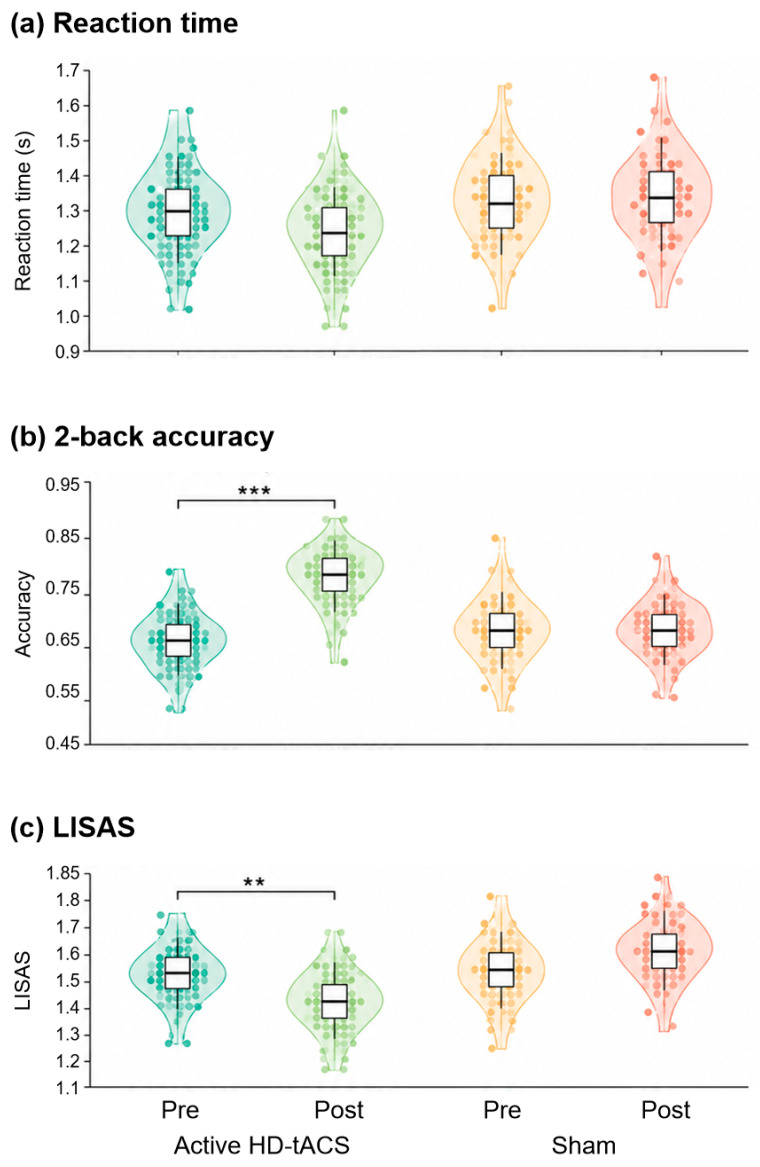
Two-back working-memory performance in Experiment 1. (**a**) Reaction time, (**b**) accuracy, and (**c**) Linear Integrated Speed–Accuracy Score (*LISAS*) before and after stimulation. In each panel, the left pair represents the active HD-tACS group and the right pair represents the sham group. Colored dots represent individual participants; violin width represents the distribution density; and white boxplots show the median (central line) and interquartile range. Lower reaction time and *LISAS* values, and higher accuracy values, indicate better performance. Brackets and asterisks indicate significant within-group pre–post follow-up comparisons. ** *p* < 0.01, *** *p* < 0.001.

These results indicate that active HD-tACS improved working-memory performance, primarily reflected by higher two-back accuracy and lower *LISAS* (Figure 6).

#### 2.2.4. Associations Between Working-Memory Improvement and Smartphone-Task Improvement

To examine whether individual differences in working-memory improvement were associated with improvements in smartphone-task performance, improvement scores were computed within the active HD-tACS group. Completion-time improvement and slope improvement were coded as pre-stimulation minus post-stimulation, whereas target-selection-accuracy improvement was coded as post-stimulation minus pre-stimulation. Thus, larger positive values consistently indicated greater improvement.

Pearson correlations were calculated between two-back improvement indices and smartphone-task improvement indices, with Benjamini–Hochberg FDR correction applied across the predefined set of correlations between two-back improvement indices and smartphone-task improvement indices. After FDR correction, improvement in two-back accuracy was significantly associated with improvement in high-difficulty task completion time, *r*(18) = 0.625, raw *p* = 0.003, FDR-adjusted *p* = 0.044, 95% CI [0.252, 0.836]. Greater improvement in two-back accuracy was also associated with a greater reduction in the difficulty–time slope, *r*(18) = 0.683, raw *p* < 0.001, FDR-adjusted *p* = 0.024, 95% CI [0.344, 0.864]. Correlations involving smartphone-task target selection accuracy improvement did not survive FDR correction.

**Figure 7:**
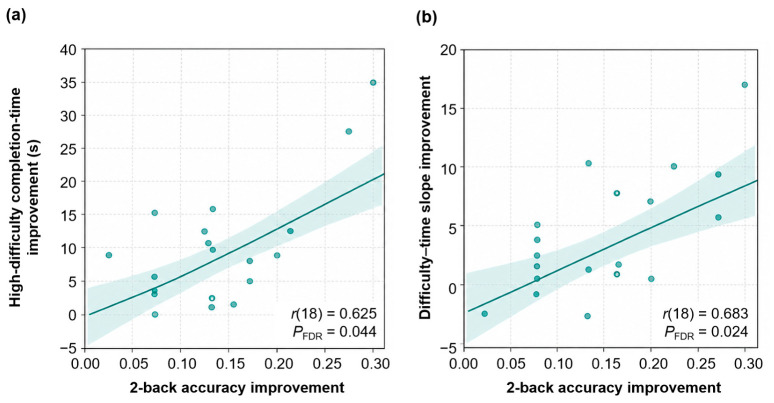
Associations between two-back accuracy improvement and smartphone-task efficiency improvement in the active HD-tACS group (*n* = 20). (**a**) Association between two-back accuracy improvement and high-difficulty completion-time improvement. (**b**) Association between two-back accuracy improvement and difficulty–time slope improvement. Each point represents one participant. Solid lines represent least-squares regression fits, and shaded bands represent 95% confidence intervals. Larger positive values indicate greater improvement. Reported *p*-values are FDR-adjusted across the predefined correlation family.

These results suggest that working-memory gains were primarily associated with improved smartphone-task efficiency, particularly under high task demands, rather than with broad improvements across all target selection accuracy indices (Figure 7).

### 2.3. Discussion

Experiment 1 provides short-term behavioral evidence that CP5-centered parietal HD-tACS is associated with improved performance in a controlled smartphone-based shopping task and improved working-memory performance. Relative to sham stimulation, active HD-tACS improved task performance in three ways. First, it reduced completion time under the high-difficulty condition, where participants were required to maintain and search for five target products. Second, it increased target selection accuracy across task-difficulty levels. Third, it reduced the difficulty–time slope, indicating that participants’ completion time became less sensitive to increasing task demands after stimulation. Together, these findings suggest that parietal neuromodulation may support selected working-memory and attentional-control components of cognitively demanding smartphone-task performance, particularly when the task requires maintenance and updating of goal-relevant information.

These results are theoretically consistent with research showing that older adults’ technology use is shaped not only by access and attitudes but also by cognitive abilities, self-efficacy, anxiety, and task demands, highlighting the need to consider age-related cognitive changes in interface design and training programs [2,10]. The present task required participants to maintain multiple target products, search through visually similar items, update selections, and verify the cart before submission. Thus, the shopping paradigm provided an ecologically oriented but experimentally controlled model of selected human–smartphone interaction operations rather than a consumer-behavior task. The finding that completion-time improvement was strongest in the high-difficulty condition further suggests that HD-tACS benefits may be most apparent when smartphone operations place higher demands on working memory and cognitive control.

Baseline-change analyses indicated that some pre–post improvements were related to initial performance. Participants with poorer baseline efficiency or lower baseline target selection accuracy tended to show larger gains in several conditions, suggesting that baseline ability, room for improvement, regression-to-the-mean, procedural familiarity, and strategy optimization may have contributed to the observed pre–post changes. The sham groups partially mitigate this concern, but they do not fully eliminate potential retest or session-related effects. Importantly, high-difficulty target selection accuracy showed no baseline ceiling effect, indicating that the high-demand target-selection-accuracy outcome retained sufficient sensitivity to detect improvement. Nevertheless, because alternate stimulus sets were not independently validated for equivalence and neither experiment used a crossover design, the contribution of stimulus-set differences and retest-related learning cannot be fully ruled out (Supplementary Table S9).

Experiment 1 also showed that active HD-tACS improved two-back working-memory performance. Specifically, participants in the active stimulation group exhibited a robust increase in two-back accuracy and a decrease in *LISAS*, whereas the sham group did not show comparable improvement. The two-back task is widely used to assess working-memory updating and monitoring processes, and meta-analytic work has shown that N-back performance engages a distributed frontoparietal working-memory network [21]. The use of *LISAS* further allowed us to account for potential speed–accuracy trade-offs, which is important in older adult samples where changes in response speed and accuracy may not move in the same direction [45]. In contrast, *RT* effects were weaker and should be interpreted cautiously because the Group × Time interaction was only marginal, and baseline *RT* showed a trend-level group difference. Therefore, the most reliable evidence for working-memory enhancement comes from two-back accuracy and *LISAS*.

The observed working-memory improvement is also consistent with prior neuromodulation research linking rhythmic stimulation of frontoparietal or parietal networks to memory enhancement. Repetitive tACS targeting memory-related cortical networks has been shown to improve working-memory performance in older adults [38], and other work suggests that synchronizing rhythmic brain circuits can restore aspects of working-memory function in aging [37]. Studies in younger adults and mixed samples further indicate that externally induced frontoparietal synchronization can modulate network dynamics and enhance working-memory performance [36], while EEG-informed theta-frequency tACS has been linked to memory-capacity changes and frontoparietal theta connectivity [39]. Although Experiment 1 did not record EEG, the behavioral pattern is compatible with this literature: CP5-centered parietal stimulation was associated with improved two-back working-memory performance and selected outcomes in a controlled smartphone-based shopping task. This behavioral pattern motivated Experiment 2, which examined whether HD-tACS-related smartphone-task changes were accompanied by task-related oscillatory modulation.

The correlation analyses further clarified the behavioral link between working-memory enhancement and smartphone-task improvement. After FDR correction, greater improvement in two-back accuracy was associated with greater improvement in high-difficulty task completion time and greater reduction in the difficulty–time slope. These associations suggest that individuals who benefited more from stimulation in working memory also tended to show greater gains in smartphone-task efficiency. Importantly, working-memory improvement was not reliably associated with target selection accuracy after correction. Thus, the present findings support a more specific interpretation: HD-tACS-related working-memory gains were associated with greater efficiency in completing cognitively demanding smartphone operations, rather than uniformly improving all behavioral indices. This distinction is important for interpreting Experiment 2, where the strongest stimulation-specific behavioral effect emerged for target selection accuracy under the reduced-trial EEG protocol.

The distinction between efficiency and target selection accuracy is theoretically meaningful. In the present paradigm, target selection accuracy reflected the proportion of instructed target products correctly retained in the submitted response. Because the submitted response was constrained to contain exactly the required number of products, a retained distractor necessarily displaced an instructed target product; thus, lower target selection accuracy also reflected a higher final distractor selection rate. Completion time and difficulty–time slope, by contrast, reflected how efficiently participants maintained the target set and navigated the task structure as cognitive load increased. Working-memory enhancement may therefore reduce the need for repeated checking, re-searching, or hesitation during high-load trials, leading to faster completion without necessarily producing equivalent gains in all target-selection-accuracy indices. This interpretation is consistent with the idea that cognitive support may be particularly useful for older adults when an interface requires sequential operations and the temporary maintenance of multiple task goals.

Several methodological considerations should be noted. First, target selection accuracy in the smartphone task reflected the correctness of the final submitted selection under a fixed-cardinality rule: participants were required to submit exactly the same number of products as instructed targets, and each retained distractor necessarily displaced one target product. Thus, the measure captured both correct target retention and final distractor selection in a single algebraically constrained index. However, the available logs did not retain the process-level sequence of temporary distractor selections, deselections, cart corrections, or error-recovery behavior before final submission. Second, because the task modeled selected smartphone-shopping operations in an ecologically oriented but experimentally controlled task, the findings should be interpreted as evidence for cognitively demanding smartphone-task performance rather than general smartphone proficiency. Third, future studies could extend the present paradigm by recruiting larger samples and measuring participants’ prior smartphone proficiency, technology anxiety, and mobile self-efficacy to further evaluate ecological validity and translational potential.

In summary, Experiment 1 provides short-term behavioral evidence that CP5-centered parietal HD-tACS was associated with improved performance in selected outcomes of a controlled smartphone-based shopping task and with improved two-back working-memory performance. The associations between two-back improvement and high-demand smartphone-task efficiency are compatible with a working-memory/control account, but do not establish that working-memory change mediated the stimulation effect. These behavioral findings provided the rationale for Experiment 2, which examined exploratory post-stimulation EEG correlates under a reduced-trial protocol.

## 3. Experiment 2: Reduced-Trial EEG Extension of the Smartphone-Based Shopping Task

Experiment 2 extended the behavioral work by incorporating EEG during smartphone-task performance. Because the number of trials was reduced to accommodate EEG recording and reduce participant burden, Experiment 2 should be interpreted as a smaller reduced-trial EEG extension rather than a full behavioral replication of Experiment 1.

### 3.1. Materials and Methods

#### 3.1.1. Participants

Twenty-eight older adults aged 60 years or above were recruited from the local community. The eligibility criteria were the same as those used in Experiment 1: participants were required to be physically and mentally healthy, right-handed, have normal or corrected-to-normal vision, report no hearing impairment, and have daily experience using smartphones. Participants were randomly assigned to either the active HD-tACS group or the sham stimulation group. The active HD-tACS group included 15 participants, six men and nine women, and the sham stimulation group included 13 participants, five men and eight women. The two groups did not differ significantly in age (*t*(26) = 1.663, *p* = 0.108), or sex distribution (χ^2^(1) = 0.007, *p* = 0.934). All participants provided written informed consent before participation, and the same safety-screening criteria as in Experiment 1 were applied.

#### 3.1.2. Design and Smartphone-Task Paradigm

Experiment 2 used a reduced-trial version of the controlled smartphone-based shopping paradigm described in Experiment 1. The task modeled cognitively demanding smartphone interaction through product search, target selection, cart checking, and final submission. As in Experiment 1, task difficulty was manipulated by varying the number of target products: three targets in the low-difficulty condition, four targets in the medium-difficulty condition, and five targets in the high-difficulty condition. For each trial, participants were required to submit exactly the same number of products as the number of instructed target products.

The same behavioral indices and scoring procedures as in Experiment 1 were used: completion time, target selection accuracy, and difficulty–time slope. Briefly, completion time indexed task efficiency, target selection accuracy indexed the proportion of instructed target products correctly retained in the submitted response under the fixed-cardinality rule, and the difficulty–time slope indexed the extent to which completion time increased with task difficulty. To reduce participant burden during EEG recording, each difficulty condition included 10 trials, yielding 30 trials in total at each measurement time.

The behavioral design was a 2 × 2 × 3 mixed design for completion time and target selection accuracy, with stimulation group as the between-subjects factor and measurement time and task difficulty as within-subject factors. Difficulty–time slope was analyzed using a 2 × 2 mixed design, with stimulation group as the between-subjects factor and measurement time as the within-subject factor.

#### 3.1.3. Procedure

The procedure of Experiment 2 is illustrated in Figure 4b. Each participant completed Experiment 2 in a single experimental session while EEG was recorded during smartphone-task performance. Participants first completed practice trials of the smartphone task for approximately 5 min, followed by a 6 min break. They then completed the pre-stimulation smartphone task while EEG was recorded. After the pre-stimulation task, participants received 20 min of active CP5-centered parietal HD-tACS or sham stimulation. Immediately after stimulation, participants completed the post-stimulation smartphone task using a new set of product stimuli while EEG was again recorded. A final 6 min break was provided before debriefing.

#### 3.1.4. HD-tACS Protocol

The HD-tACS protocol was identical to that used in Experiment 1 and used the same CP5-centered 4 × 1 montage shown in Figure 3. Briefly, stimulation was delivered at 4 Hz for 20 min with a peak current intensity of 1.5 mA. The 4 Hz frequency was selected to target theta-band activity, given prior evidence linking theta-band synchronization and theta-frequency tACS to working-memory and memory-related processes [36,37,39]. The sham condition used the same ramp-up/ramp-down procedure as in Experiment 1 to mimic the initial sensory experience without delivering sustained alternating current.

#### 3.1.5. EEG Acquisition and Preprocessing

EEG was recorded using a SynAmps RT 64-channel amplifier system with Curry 9 software (Compumedics USA, Inc., Charlotte, NC, USA). Before data acquisition, EEG electrode impedances were kept below 5 kΩ. Continuous EEG signals were recorded at a sampling rate of 1000 Hz from 16 electrode sites based on the international 10–10 system: AF3, F5, F3, F1, FT7, FC5, FC3, FC1, TP7, CP5, CP3, CP1, P5, P3, P1, and PO3. FCz served as the online reference.

EEG preprocessing was conducted in MATLAB R2023a (MathWorks, Natick, MA, USA) using EEGLAB (version 2025.0.0; Swartz Center for Computational Neuroscience, La Jolla, CA, USA) [48]. The continuous data were band-pass filtered from 0.1 to 60 Hz, down-sampled to 500 Hz, and notch-filtered from 48 to 52 Hz to reduce power-line noise. The data were segmented into 4 s epochs. Epochs containing prominent motion artifacts, eye movements, eye blinks, or voltage amplitudes exceeding ±100 μV were rejected. Bad channels were interpolated using spherical-spline interpolation when necessary. The data were then re-referenced to the average of the bilateral mastoids.

Independent component analysis (ICA) was used to identify and remove ocular and muscle-related artifacts. Independent components were labeled using the ICLabel plugin (version 1.6; Swartz Center for Computational Neuroscience, La Jolla, CA, USA), and components classified with high probability as eye-movement or muscle artifacts were removed [49]. On average, preprocessing resulted in the rejection of 29.26% of epochs per participant, *SD* = 20.05%.

#### 3.1.6. EEG Power Analysis

Power spectral density (PSD) was computed for each preprocessed epoch using Fast Fourier Transform. Mean PSD values were extracted for four frequency bands: theta, 4–8 Hz; alpha, 8–12 Hz; beta, 12–30 Hz; and gamma, 30–60 Hz. Theta and alpha oscillations have been widely linked to cognitive and memory-related processes, while theta-band activity has also been implicated in cognitive control and task monitoring [31,32,34].

For ROI-level analyses, the 16 electrodes were grouped into five regions of interest: frontal ROI, including AF3, F1, F3, and F5; central ROI, including FC1, FC3, and FC5; temporal ROI, including FT7 and TP7; IPL ROI, including P3, P5, CP3, and CP5; and posterior parieto-occipital ROI, including P1, CP1, and PO3. Mean PSD was calculated for each frequency band, ROI, measurement time, and participant.

For each frequency band, EEG power was analyzed using a 2 × 2 × 5 mixed-design ANOVA, with stimulation group as the between-subjects factor and measurement time and ROI as within-subject factors. Theta-band activity was the a priori frequency band of interest. Therefore, theta-related follow-up analyses were treated as hypothesis-guided. However, when the relevant omnibus interaction did not reach conventional significance, these follow-up analyses were interpreted as exploratory rather than confirmatory. Greenhouse–Geisser correction was applied when the sphericity assumption was violated.

#### 3.1.7. EEG–Behavior Correlation Analysis

To examine whether EEG power changes were associated with behavioral improvement, change scores were computed within the active HD-tACS group. Completion-time improvement and slope improvement were coded as pre-stimulation minus post-stimulation. Target selection accuracy improvement was coded as post-stimulation minus pre-stimulation. Theta-power change was coded as post-stimulation minus pre-stimulation.

For EEG–behavior correlations in Experiment 2, we distinguished between a theory-defined ROI family and a broader exploratory family. The theory-defined family tested correlations between high-demand target-selection-accuracy improvement and theta-power changes across the five predefined ROIs in the active-stimulation group, with FDR correction applied across these five tests. To transparently evaluate the broader exploratory pattern, we also report the full 45-test matrix crossing nine behavioral improvement indices with five theta ROIs, with FDR correction applied across all 45 tests. The theory-defined analyses were interpreted as hypothesis-guided exploratory evidence, whereas the full 45-test matrix was used to evaluate the robustness of the broader correlation pattern.

### 3.2. Results

Descriptive statistics for smartphone-task performance and theta-band ROI power in Experiment 2 are reported in Table 3 and Table 4, respectively. Target selection accuracy was defined as the proportion of instructed target products correctly retained in the submitted response. Lower completion time and lower difficulty–time slope indicate better performance, whereas higher target selection accuracy indicates better performance. EEG power values in Table 4 indicate mean theta-band PSD values for each ROI. Descriptive statistics for all EEG frequency bands are provided in Supplementary Table S2.

#### 3.2.1. Baseline Comparisons

At baseline, the active HD-tACS and sham stimulation groups did not differ significantly in smartphone-task completion time, target selection accuracy, or difficulty–time slope, all *ps* > 0.09. Thus, subsequent behavioral analyses focused on Group × Time interactions and pre-to-post changes.

#### 3.2.2. Effects of HD-tACS on Smartphone-Task Performance

##### Completion Time

For smartphone-task completion time, the main effect of task difficulty was significant (*F*(2, 52) = 92.143, *p* < 0.001, η^2^_p_ = 0.780), indicating that participants required more time as the number of target products increased. The main effect of measurement time was also significant (*F*(1, 26) = 9.104, *p* = 0.006, η^2^_p_ = 0.259), indicating an overall reduction in completion time from pre- to post-stimulation. The main effect of the stimulation group was not significant (*F*(1, 26) = 0.304, *p* = 0.586, η^2^_p_ = 0.012).

The Group × Difficulty interaction was significant (*F*(2, 52) = 3.281, *p* = 0.045, η^2^_p_ = 0.112), suggesting that the two groups differed in the extent to which completion time increased with task difficulty. However, the critical Group × Time interaction was not significant (*F*(1, 26) = 0.925, *p* = 0.345, η^2^_p_ = 0.034), and the Group × Time × Difficulty interaction was also not significant (*F*(2, 52) = 0.330, *p* = 0.720, η^2^_p_ = 0.013). Therefore, Experiment 2 did not provide confirmatory evidence for a stimulation-specific improvement in completion time.

##### Target Selection Accuracy

For target selection accuracy, the main effect of task difficulty was significant (*F*(2, 52) = 164.979, *p* < 0.001, η^2^_p_ = 0.864), indicating lower target selection accuracy as task difficulty increased. The main effect of measurement time was significant (*F*(1, 26) = 26.967, *p* < 0.001, η^2^_p_ = 0.509), reflecting an overall increase in target selection accuracy from pre- to post-stimulation. The main effect of the stimulation group was not significant (*F*(1, 26) < 0.001, *p* = 0.991, η^2^_p_ < 0.001).

Importantly, the Group × Time interaction was significant (*F*(1, 26) = 9.149, *p* = 0.006, η^2^_p_ = 0.260). The Time × Difficulty interaction showed a trend (*F*(2, 52) = 2.721, *p* = 0.075, η^2^_p_ = 0.095). The Group × Difficulty interaction and the Group × Time × Difficulty interaction were not significant (both *ps* > 0.33). Follow-up comparisons showed that the active HD-tACS group exhibited a robust overall increase in target selection accuracy after stimulation (*t*(14) = 6.741, *p* < 0.001, *d*z = 1.740), whereas the sham stimulation group did not show a significant overall change (*t*(12) = 1.169, *p* = 0.265, *d*z = 0.324).

Difficulty-specific follow-up analyses suggested that, within the active HD-tACS group, target selection accuracy increased after stimulation in the medium-difficulty and high-difficulty conditions after Bonferroni correction, with the strongest improvement observed in the high-difficulty condition. Because the Group × Time × Difficulty interaction was not significant, these difficulty-specific comparisons should be interpreted as descriptive rather than confirmatory evidence for difficulty-dependent stimulation effects.

##### Difficulty–Time Slope

For the difficulty–time slope, the main effect of measurement time was significant (*F*(1, 26) = 6.432, *p* = 0.018, η^2^_p_ = 0.198), indicating that the difficulty–time slope decreased overall from pre- to post-stimulation. The main effect of the stimulation group showed a trend (*F*(1, 26) = 3.600, *p* = 0.069, η^2^_p_ = 0.122). However, the Group × Time interaction was not significant (*F*(1, 26) = 0.654, *p* = 0.426, η^2^_p_ = 0.025). Thus, Experiment 2 did not provide evidence for a stimulation-specific reduction in the difficulty–time slope.

**Figure 8:**
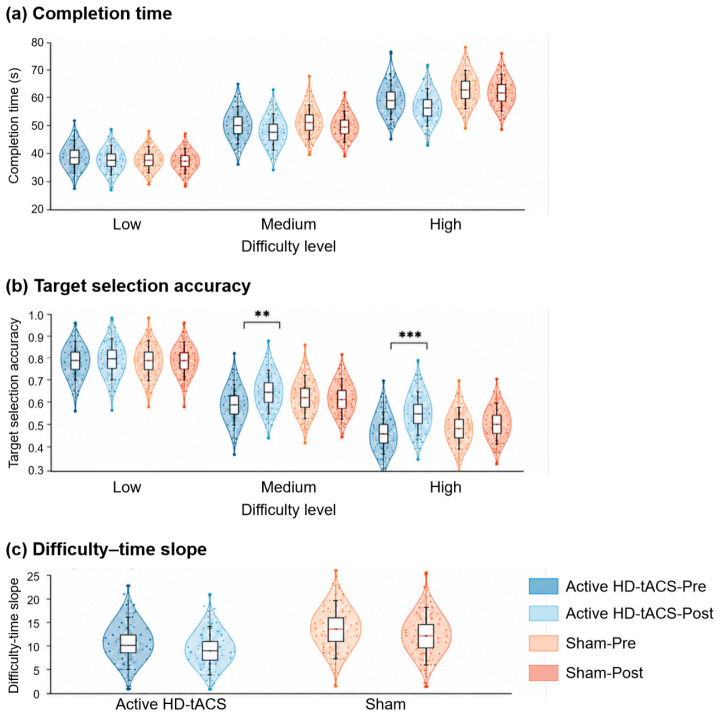
Smartphone-task performance in Experiment 2. (**a**) Completion time across low-, medium, and high-difficulty conditions. (**b**) Target selection accuracy across difficulty conditions. (**c**) Difficulty–time slope. Colored dots represent individual participants; violin width represents the distribution density; and white boxplots show the median (central line) and interquartile range. Colors distinguish the active HD-tACS and sham groups at pre- and post-stimulation. Brackets and asterisks in panel (**b**) indicate exploratory within-group pre–post follow-up comparisons in the active HD-tACS group. Because the Group × Time × Difficulty interaction was not significant, these difficulty-specific follow-up results should be interpreted cautiously. ** *p* < 0.01, *** *p* < 0.001.

The behavioral effects of HD-tACS in Experiment 2 are shown in Figure 8. Together, the behavioral results of Experiment 2 provide complementary evidence for the effect of HD-tACS on smartphone-task performance. The most robust stimulation-specific behavioral effect was observed for target selection accuracy, suggesting improved goal maintenance and target identification within the controlled smartphone-based shopping task. Completion time and difficulty–time slope showed general pre-to-post improvements but did not show stimulation-specific interaction effects, indicating that efficiency-related effects were less stable under the reduced-trial EEG protocol than in Experiment 1.

#### 3.2.3. EEG Data-Quality Check

EEG data-quality indices were computed from the preprocessed EEG datasets using the common 16-channel set included in the EEG power analyses. The proportion of rejected epochs showed a significant Group × Time interaction, *F*(1, 26) = 5.109, *p* = 0.032. In the active group, the rejected-epoch proportion increased from pre-stimulation (*M* = 0.280, *SD* = 0.188) to post-stimulation (*M* = 0.358, *SD* = 0.232). In the sham group, the rejected-epoch proportion decreased from pre-stimulation (*M* = 0.306, *SD* = 0.221) to post-stimulation (*M* = 0.225, *SD* = 0.152). We therefore conducted additional sensitivity analyses to examine whether theta-power results were robust to data-quality variation. ANCOVA models adjusting for baseline theta power and change in rejected-epoch proportion indicated that group-level theta-power changes were no longer statistically reliable after accounting for EEG data quality. The active-group IPL theta–behavior association remained significant when controlling for rejected-epoch change. In a complementary sensitivity analysis excluding participants with more than 50% rejected epochs in either EEG session, the association remained positive but was no longer statistically significant after FDR correction (Supplementary Table S11). Together, these results indicate that EEG findings should be interpreted as preliminary post-stimulation associations rather than as definitive evidence for stimulation-induced theta modulation (Supplementary Table S11; Supplementary Figure S2).

Because this interaction indicates that EEG data quality varied across sessions and groups, EEG findings should be interpreted cautiously. Importantly, this issue does not directly account for the behavioral target-selection-accuracy effect, but it should be considered when interpreting stimulation-related changes in oscillatory power.

#### 3.2.4. Effects of HD-tACS on EEG Power

##### Theta-Band Power

For theta-band power, the main effect of measurement time was significant (*F*(1, 26) = 7.609, *p* = 0.010, η^2^_p_ = 0.226), indicating an overall increase in theta power from pre- to post-stimulation. The main effect of ROI was significant (*F*(4, 104) = 63.595, *p* < 0.001, η^2^_p_ = 0.710), reflecting regional variation in theta power. The Time × ROI interaction was also significant (*F*(4, 104) = 2.972, *p* = 0.023, η^2^_p_ = 0.103).

The critical Group × Time interaction showed a trend but did not reach conventional significance (*F*(1, 26) = 3.875, *p* = 0.060, η^2^_p_ = 0.130). The Group × Time × ROI interaction also showed a trend (*F*(4, 104) = 2.354, *p* = 0.059, η^2^_p_ = 0.083). The theta-band results therefore provide preliminary evidence of post-stimulation theta-power changes following HD-tACS, although the stimulation-specific effect should be interpreted cautiously because the Group × Time interaction reached trend-level significance rather than conventional significance.

Given the a priori focus on theta-band activity and the trend-level Group × Time and Group × Time × ROI interactions, hypothesis-guided exploratory follow-up analyses were conducted. At the whole-ROI-mean level, theta power increased significantly from pre- to post-stimulation in the active HD-tACS group (*t*(14) = 2.783, *p* = 0.015, *d*z = 0.719), but not in the sham stimulation group (*t*(12) = 0.643, *p* = 0.533, *d*z = 0.178). ROI-specific exploratory comparisons suggested theta increases in the active HD-tACS group, with the most robust effects observed in posterior regions, including the IPL and posterior parieto-occipital ROI.

##### Alpha-, Beta-, and Gamma-Band Power

For alpha-band power, the main effect of measurement time was significant (*F*(1, 26) = 12.036, *p* = 0.002, η^2^_p_ = 0.316), indicating an overall increase in alpha power from pre- to post-stimulation. The main effect of ROI was significant (*F*(4, 104) = 23.345, *p* < 0.001, η^2^_p_ = 0.473), and the Time × ROI interaction was also significant (*F*(4, 104) = 3.667, *p* = 0.008, η^2^_p_ = 0.124). However, the Group × Time interaction was not significant (*F*(1, 26) = 1.967, *p* = 0.173, η^2^_p_ = 0.070), and the Group × Time × ROI interaction was also not significant (*F*(4, 104) = 0.938, *p* = 0.445, η^2^_p_ = 0.035). Thus, alpha-band changes were robust at the session level but were not specific to active HD-tACS.

For beta-band power, neither the main effect of measurement time nor the Group × Time interaction was significant (both *ps* > 0.52). The main effect of ROI was significant (*F*(4, 104) = 23.854, *p* < 0.001, η^2^_p_ = 0.478), indicating regional variation in beta power. For gamma-band power, neither the main effect of measurement time nor the Group × Time interaction was significant (both *ps* > 0.64). The main effect of ROI was significant (*F*(4, 104) = 41.246, *p* < 0.001, η^2^_p_ = 0.613).

**Figure 9:**
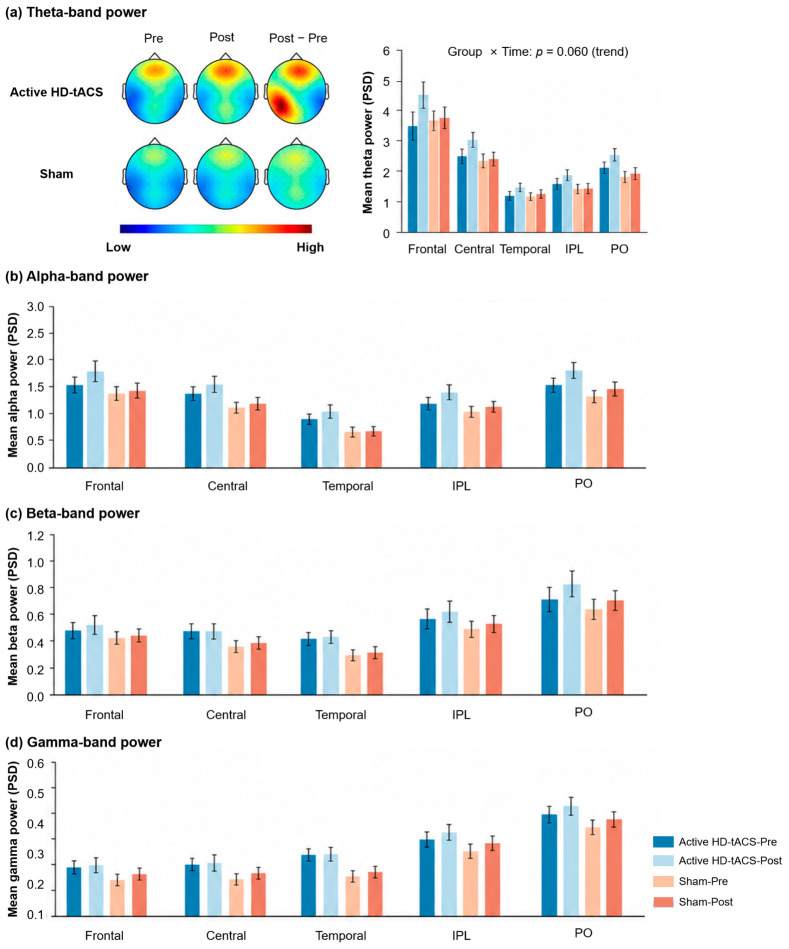
Task-related EEG power by frequency band in Experiment 2. (**a**) Theta-band power. The left side shows group-level scalp topographies of mean theta power before stimulation, after stimulation, and for the post–pre change, using a shared relative color scale. The right side shows ROI-level mean theta power. (**b**–**d**) ROI-level mean alpha-, beta-, and gamma-band power, respectively. Bars show group means, and error bars show standard errors of the mean. Colors distinguish the active HD-tACS and sham groups at pre- and post-stimulation. The Group × Time effect for theta power was trend-level (*p* = 0.060) and should be interpreted as preliminary. Alpha power showed a session-level increase without a stimulation-specific interaction; no stimulation-specific effects were observed for beta or gamma power. No ROI-specific significance markers are shown because follow-up comparisons were exploratory. PO = posterior parieto-occipital ROI.

Overall, EEG power analyses showed a significant general increase in theta power and trend-level evidence for possible stimulation-related theta modulation. Alpha power also increased over time, but this effect was not specific to active stimulation. No stimulation-specific effects were observed in beta or gamma power (Figure 9).

#### 3.2.5. Associations Between Theta-Power Changes and Smartphone-Task Improvement

To test whether theta-band modulation was associated with behavioral improvement, Pearson correlations were computed within the active HD-tACS group between high-difficulty target selection accuracy improvement and theta-power change across the five ROIs. This analysis was selected as the primary theory-defined correlation family because Experiment 1 indicated that stimulation effects were most evident under higher task demands, and theta-band activity was the a priori EEG frequency of interest.

After FDR correction across the five ROI-level tests, high-difficulty target selection accuracy improvement was significantly associated with theta-power changes in the temporal ROI (*r*(13) = 0.635, raw *p* = 0.011, FDR-adjusted *p* = 0.018); IPL ROI (*r*(13) = 0.747, raw *p* = 0.001, FDR-adjusted *p* = 0.007); and posterior parieto-occipital ROI (*r*(13) = 0.663, raw *p* = 0.007, FDR-adjusted *p* = 0.018). Correlations with the frontal and central ROIs did not reach significance after FDR correction. These hypothesis-guided correlations are reported in Table 5.

**Figure 10:**
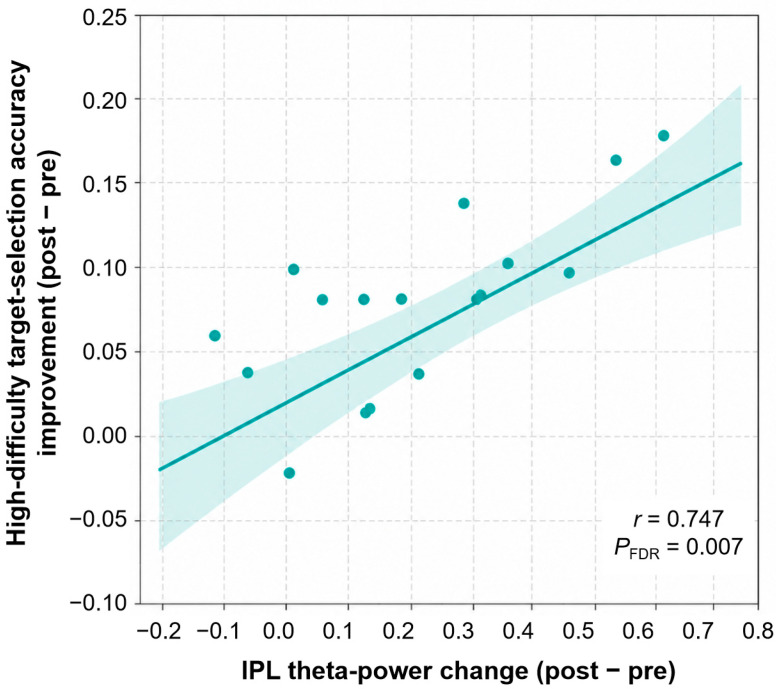
Association between IPL theta-power change and high-difficulty target selection accuracy improvement in the active HD-tACS group (*n* = 15). Each point represents one participant. The solid line represents the least-squares regression fit, and the shaded band represents the 95% confidence interval. Theta-power change and target selection accuracy improvement were calculated as post-stimulation minus pre-stimulation. The reported *p*-value is FDR-adjusted across the five theory-defined ROI-level tests. This hypothesis-guided EEG–behavior association should be interpreted as exploratory because the relevant omnibus EEG effects were trend-level.

For transparency, we also examined the full exploratory matrix of 45 ROI-level EEG–behavior correlations, which is reported in Supplementary Table S1. None of the correlations survived FDR correction across the full matrix at q < 0.05. Therefore, the EEG–behavior findings should be interpreted as hypothesis-guided exploratory evidence that theta-power changes in posterior regions, particularly the IPL ROI, were associated with high-demand target-selection-accuracy improvement, rather than as broad evidence that theta changes predicted all behavioral outcomes. The key association between IPL theta-power change and high-difficulty target selection accuracy improvement is shown in Figure 10.

### 3.3. Discussion

Experiment 2 provided a reduced-trial EEG extension of the behavioral findings, with exploratory post-stimulation EEG associations during performance in a controlled smartphone-based shopping task. Behaviorally, the most robust stimulation-specific effect emerged for target selection accuracy, which may reflect changes in goal maintenance and target identification within the present task. Completion time and difficulty–time slope showed general pre-to-post improvements but did not show significant Group × Time interactions, indicating that efficiency-related effects were less stable under the reduced-trial EEG protocol than in Experiment 1. This pattern suggests that Experiment 2 should be interpreted primarily as an EEG extension of the behavioral findings rather than as an exact replication of all behavioral effects observed in Experiment 1.

The pattern of behavioral findings is informative for interpreting the role of cognitive demand in smartphone-task performance. In Experiment 1, active HD-tACS improved completion time most clearly under the high-difficulty condition and reduced the difficulty–time slope. In Experiment 2, where the number of trials was reduced to accommodate EEG recording, the most reliable behavioral effect was observed in target selection accuracy. This suggests that the EEG protocol may have been more sensitive to changes in goal maintenance and target identification than to stable estimates of completion-time efficiency. Importantly, target selection accuracy captures a core component of human–smartphone interaction in the present paradigm: the ability to maintain task goals and identify relevant targets within a visually complex interface.

At the neural level, theta-band power showed a significant overall increase from pre- to post-stimulation, and the Group × Time interaction showed a trend-level effect. Follow-up analyses suggested theta-power increases in the active HD-tACS group, particularly in posterior regions including the IPL. However, because the critical Group × Time interaction did not reach conventional significance, these EEG effects should be interpreted as exploratory post-stimulation evidence of theta-power changes rather than as confirmatory evidence of stimulation-induced entrainment. This distinction is important because EEG was recorded before and after stimulation, not during stimulation; therefore, the present data speak to post-stimulation task-related oscillatory changes rather than online entrainment itself.

The theta-band finding is nevertheless consistent with a broader literature linking theta oscillations to memory and cognitive control. Theta activity has been associated with memory processes and task-related cognitive control [31,32,34]. In the context of tACS, externally modulating rhythmic brain activity has been shown to influence working-memory performance and frontoparietal network dynamics [36,37,39]. The present results extend this literature by examining oscillatory changes during a controlled smartphone-based shopping task in older adults. Rather than demonstrating entrainment directly, the findings suggest that theta-band modulation may accompany improvements in cognitively demanding smartphone interaction, particularly under high task-demand conditions consistent with the behavioral pattern observed in Experiment 1.

Alpha-band power also increased from pre- to post-stimulation, with regional variation across ROIs. Although this effect was not specific to active HD-tACS, it may still reflect general task-related attentional adaptation. Alpha oscillations have been linked to attentional allocation, functional inhibition of task-irrelevant processing, and memory-related information control [32,34,50,51]. In the present controlled smartphone-based shopping task, increased alpha power may reflect session-level changes in attentional stabilization, visual information filtering, or task familiarization as participants repeated the smartphone task. Therefore, alpha-band changes should be interpreted as a general task-related oscillatory adaptation rather than as a selective neural signature of active HD-tACS.

The EEG–behavior correlation results provide a more targeted, but exploratory, association between theta-power change and behavioral improvement. In the active HD-tACS group, high-difficulty target-selection-accuracy improvement was associated with theta-power increases in posterior regions, including the IPL ROI. This pattern is compatible with the involvement of posterior/parietal regions in visual attention and working-memory-related control under high task demands. However, these correlations were based on a small active-stimulation subgroup, followed trend-level omnibus EEG effects, and no correlation survived FDR correction across the full exploratory 45-test matrix. Accordingly, these findings are interpreted as hypothesis-guided exploratory post-stimulation EEG associations rather than as confirmatory evidence that theta modulation mediated behavioral improvement.

The EEG data-quality analysis also suggests caution in interpreting neural effects. Although standard artifact-rejection and ICA procedures were used, the proportion of rejected epochs showed a Group × Time interaction. This pattern highlights the importance of considering data quality when interpreting pre-to-post EEG power changes in stimulation studies. Future studies would benefit from incorporating richer trial-level EEG quality indices, balanced artifact-control procedures, and larger samples to determine whether theta-band modulation remains robust after accounting for data-quality variation.

Taken together, Experiment 2 should be interpreted as a reduced-trial EEG extension rather than a full behavioral replication of Experiment 1. The most robust behavioral effect in this protocol was observed for target selection accuracy, whereas efficiency-related measures were less stable, probably because the number of trials was reduced to accommodate EEG recording and reduce participant burden. The EEG findings provide hypothesis-guided exploratory evidence that post-stimulation theta-power changes in posterior/parietal regions may accompany high-demand target-selection-accuracy improvement. However, the critical EEG omnibus effects were only trend-level, EEG data-quality indices differed across sessions and groups, and the active-group EEG–behavior correlations were based on a small sample. Therefore, the present data do not demonstrate online entrainment, mediation, or a definitive neural mechanism. They should instead be viewed as preliminary post-stimulation EEG associations that motivate larger, better-powered studies with stronger artifact-control procedures and MRI-guided, participant-specific stimulation targeting.

## 4. General Discussion

The present study examined whether CP5-centered parietal HD-tACS was associated with short-term changes in older adults’ performance in a controlled smartphone-based shopping task and whether behavioral changes were accompanied by exploratory post-stimulation EEG correlates. Experiment 1 provided the main behavioral test: relative to sham stimulation, active stimulation was associated with reduced high-demand completion time, improved target selection accuracy, a reduced difficulty–time slope, and improved two-back working-memory performance. Individual differences in two-back improvement were associated with high-demand completion-time improvement and reduced difficulty–time slope, but these correlations do not establish a mediating mechanism. Experiment 2 was a reduced-trial EEG extension in which the most robust stimulation-specific behavioral effect was target selection accuracy. EEG analyses showed trend-level omnibus effects and theory-defined posterior/parietal theta–behavior associations within a small active-stimulation subgroup; the full 45-test exploratory correlation matrix did not survive FDR correction. Therefore, the EEG findings are interpreted as exploratory post-stimulation associations rather than definitive neural support for the behavioral effects.

### 4.1. HD-tACS and Smartphone-Task Performance in Older Adults

A central contribution of the present study is that it examines a controlled smartphone-based shopping task that is more ecologically structured than conventional laboratory working-memory tasks. Older adults’ difficulties with digital technologies are often discussed in terms of access, attitudes, anxiety, and self-efficacy, but prior research has also emphasized that cognitive abilities and age-related changes in capacity are major contributors to technology use and digital inclusion [2,10,52]. The present findings support this cognitive interpretation of the age-related digital divide. Smartphone-task performance in our paradigm was not a simple measure of familiarity with online shopping. Rather, it required participants to maintain multiple target goals, search for visually similar product items, select relevant targets, check selected items, and submit a final response. These operations resemble broader components of everyday human–smartphone interaction, including visual search, goal maintenance, sequential action, and response verification.

Experiment 1 provided short-term behavioral evidence that CP5-centered parietal HD-tACS was associated with improved performance in selected outcomes of this cognitively demanding smartphone-based task. Active stimulation reduced completion time specifically in the high-difficulty condition, where participants had to maintain and search for the largest target set. It also increased target selection accuracy across difficulty levels and reduced the difficulty–time slope, indicating that participants’ completion time became less sensitive to increasing task demands. This pattern is theoretically meaningful because the slope measure captures the cost of increasing task difficulty rather than performance at a single difficulty level. A reduced difficulty–time slope suggests that active HD-tACS was associated with more efficient performance under increasing cognitive demands during smartphone interaction.

The behavioral pattern in Experiment 2 was more selective. Under the EEG protocol, active HD-tACS produced the most robust stimulation-specific effect on target selection accuracy, whereas completion time and difficulty–time slope showed general pre-to-post improvements without significant Group × Time interactions. This difference should not be interpreted as a failure of the overall account. Experiment 2 intentionally reduced the number of trials to accommodate EEG recording and reduce participant burden, which likely reduced the stability and statistical power of efficiency-related behavioral estimates. Completion time and slope are particularly sensitive to trial-to-trial variability, strategy shifts, and motor or interface-related hesitation. Target selection accuracy may therefore have been the more stable behavioral index under the reduced-trial EEG protocol. Taken together, the two experiments suggest that HD-tACS-related benefits may appear in different behavioral indices depending on measurement context: Experiment 1 showed robust effects on efficiency and target selection accuracy, whereas Experiment 2 provided complementary evidence that active stimulation was associated with improved target selection accuracy, which may reflect changes in goal maintenance and target identification within the present task.

This distinction is important for interpreting smartphone-task performance as a multidimensional construct. In real-world digital behavior, improved performance may involve faster completion, higher accuracy, reduced difficulty cost, fewer checking behaviors, or better speed–accuracy integration. These outcomes are related but not interchangeable. A stimulation protocol that improves working-memory-related control may reduce the cost of high cognitive demand in one context and improve target identification in another. The present results therefore support a nuanced account: CP5-centered parietal HD-tACS may be associated with improved smartphone-task performance through changes in cognitive processes involved in maintaining goals and managing task demands, but the behavioral manifestation of this facilitation may depend on task length, measurement reliability, and the specific performance index.

### 4.2. Working Memory as a Bridge Between Neuromodulation and Smartphone-Task Performance

The present findings also support the role of working memory as a bridge between neural stimulation and complex smartphone behavior. Working memory supports temporary maintenance and manipulation of task-relevant information, updating of ongoing goals, and coordination of sequential behavior [19,20]. These functions are directly relevant to smartphone shopping: users must remember what to find, distinguish targets from distractors, track selected items, and verify whether the final cart matches the task goal. These requirements become more demanding as the number of target items increases.

Experiment 1 showed that active HD-tACS improved two-back working-memory performance, primarily reflected by increased accuracy and reduced *LISAS*. The *RT* effect was only trend-level and was not treated as primary evidence, especially given the trend-level baseline *RT* difference. This pattern strengthens the interpretation that the working-memory effect was not simply a global speeding effect. The improvement in *LISAS* is particularly useful because it integrates speed and accuracy and helps account for possible speed–accuracy trade-offs. In older adults, such integrated measures can be informative because response speed and accuracy may change in different directions.

The correlation findings further support the working-memory interpretation. Within the active HD-tACS group, greater improvement in two-back accuracy was associated with greater improvement in high-difficulty completion time and greater reduction in the difficulty–time slope after FDR correction. In contrast, correlations involving smartphone-task target selection accuracy did not survive FDR correction. This pattern suggests that working-memory enhancement was most strongly related to the efficiency with which older adults managed cognitively demanding smartphone operations. One possible interpretation is that improved working memory reduced the need for repeated checking, re-searching, or hesitation when participants had to maintain multiple targets under high task load. This would lead to faster completion and a lower difficulty–time slope. Target-selection-accuracy improvements, by contrast, may depend not only on working memory but also on visual discrimination, interface familiarity, response caution, and strategy.

This interpretation is consistent with broader cognitive aging frameworks. The scaffolding theory of aging and cognition proposes that older adults’ performance depends on the interaction between age-related neural decline and compensatory recruitment of cognitive-control systems [23]. The maintenance, reserve, and compensation framework similarly emphasizes that older adults vary in their ability to maintain cognitive function and recruit compensatory mechanisms under demand [24]. From this perspective, 4 Hz CP5-centered parietal HD-tACS may have transiently supported a parietal component of the working-memory/control network, allowing participants to handle high-demand smartphone operations more efficiently. This does not imply that stimulation restored a broad or permanent cognitive capacity. Rather, it suggests that modulation of a task-relevant neural system may improve performance when the task places sufficient demand on that system.

### 4.3. Theta-Band Modulation and the Role of Posterior Regions

Experiment 2 was designed to examine whether the behavioral effects observed in Experiment 1 were accompanied by post-stimulation task-related oscillatory changes. The EEG findings were most relevant for theta-band activity. Theta oscillations have been widely linked to memory, cognitive control, task monitoring, and coordination of distributed neural activity [31,32,34,53]. In the present study, theta power increased from pre- to post-stimulation, and the Group × Time and Group × Time × ROI interactions showed trend-level effects. Exploratory follow-up analyses suggested theta increases in the active HD-tACS group, particularly in posterior regions including the IPL and posterior parieto-occipital ROI. Because the relevant omnibus effects were trend-level, these follow-up results are interpreted as hypothesis-guided exploratory analyses rather than confirmatory evidence of stimulation-induced theta modulation.

These findings are broadly consistent with the rationale for examining a CP5-centered parietal montage and posterior/parietal task-related processes. The parietal cortex is implicated in visual working memory, attentional control, and capacity-limited maintenance of goal-relevant information [22,25,54]. Smartphone-task performance in the present paradigm required participants to maintain target representations while searching and verifying information across interface states. Increased task-related theta power in posterior regions may therefore reflect greater engagement or modulation of neural processes supporting goal maintenance and target identification during complex smartphone interaction.

The EEG–behavior correlation results provide a more specific link between theta modulation and behavioral improvement. In the active HD-tACS group, high-difficulty target-selection-accuracy improvement was significantly associated with theta-power increases in the temporal, IPL, and posterior parieto-occipital ROIs after FDR correction across the theory-defined five-ROI family. The strongest association was observed in the IPL ROI. This spatial pattern is compatible with the involvement of posterior/parietal regions in visual attention and working-memory-related control; however, CP5 placement was intended only to approximate a left parietal region rather than provide anatomically precise IPL targeting. The association with posterior parieto-occipital regions also fits the visual-search demands of the task, whereas the temporal association may reflect additional involvement of object-related processing during product identification. However, these interpretations should remain cautious because the full exploratory 45-test correlation matrix did not survive FDR correction. The correlation results are best viewed as hypothesis-guided exploratory post-stimulation EEG associations rather than as confirmatory evidence that theta modulation mediated behavioral improvement.

It is important to clarify what the EEG findings do and do not show. The present design recorded EEG before and after stimulation, not during stimulation. Therefore, the data do not directly demonstrate online entrainment, phase alignment during stimulation, or causal mediation of behavioral effects by theta modulation. Instead, the findings indicate that behavioral improvement was accompanied by post-stimulation task-related oscillatory changes that may be consistent with the proposed theta-related mechanism. This distinction is essential because the tACS literature increasingly emphasizes the complexity of stimulation effects, including individual differences in endogenous oscillatory frequency, brain state, stimulation intensity, montage, and task context [28,30,55]. Thus, the present EEG results should be understood as preliminary post-stimulation EEG associations that support the plausibility of theta involvement, rather than as definitive proof of direct entrainment or causal mediation.

### 4.4. Alpha-Band Changes and Broader Oscillatory Adaptation

Although theta was the a priori frequency band of interest, Experiment 2 also showed a significant general increase in alpha power from pre- to post-stimulation. This alpha effect was not specific to active HD-tACS because the Group × Time and Group × Time × ROI interactions were not significant. Therefore, it should not be interpreted as a stimulation-specific neural mechanism. Nevertheless, the alpha result may still be informative about the task context. Alpha oscillations have been associated with attentional allocation, suppression of task-irrelevant information, and regulation of visual processing [32,34,54]. In a visually dense smartphone task, increased alpha power across sessions may reflect general task familiarization, stabilization of attentional strategy, or more efficient filtering of irrelevant visual information as participants repeated the task.

The absence of stimulation-specific alpha effects helps sharpen the interpretation of the theta findings. If all frequency bands had shown comparable stimulation-specific effects, the results would be difficult to interpret mechanistically. Instead, the strongest theoretically relevant pattern emerged in theta, while alpha reflected a broader session-level change, and beta/gamma did not show stimulation-specific effects. This profile supports a cautious frequency-specific interpretation: theta-band activity is the most plausible oscillatory candidate for the stimulation-related mechanism, whereas alpha may reflect general adaptation to repeated task performance. Future studies could examine whether theta and alpha jointly contribute to different components of smartphone-task performance, for example, by separating goal maintenance, visual filtering, and response verification phases within each trial.

### 4.5. Implications for Digital-Aging Research and Human–Technology Interaction

The present study has implications for research on digital aging and human–computer interaction. Existing approaches to reducing the age-related digital divide often emphasize interface redesign, training, technical support, and social assistance. These approaches are essential. However, the present findings suggest that cognitive capacity and neural function should also be considered when explaining why older adults differ in their ability to use smartphones effectively. If complex smartphone interaction depends partly on working memory and cognitive control, then interventions that support these functions may complement design- and training-based approaches.

This does not mean that neuromodulation should replace usability-centered design. On the contrary, the findings highlight the importance of aligning interface demands with older adults’ cognitive resources. A cognitively demanding interface may impose high working-memory load, require excessive switching between screens, or force users to remember too many intermediate goals. Age-friendly design can reduce these demands, whereas cognitive or neural interventions may be investigated as potential ways to support performance under these demands. Future digital-aging research may examine whether accessible interface design, training, and support can be integrated with cognitive and neuromodulation research, while avoiding the assumption that the present task measures digital inclusion directly.

The smartphone shopping paradigm used here also contributes methodologically. It provides an ecologically familiar but experimentally controllable context for studying older adults’ smartphone-task performance. The task is not intended to measure consumer behavior. Instead, it models core operations common to digital interaction: maintaining goals, searching visually presented information, selecting relevant options, checking intermediate outcomes, and confirming final responses. This type of task can help bridge the gap between laboratory cognitive tests and broad self-report measures of technology use. Future work could extend this paradigm to other everyday smartphone activities, such as medication management, appointment booking, mobile payment, navigation, or communication tasks.

The present smartphone-shopping paradigm was intended to balance ecological orientation and experimental control. On the one hand, product search, target selection, cart checking, and final submission are familiar operations in many smartphone-shopping contexts and therefore provide a more ecologically structured setting than conventional laboratory working-memory tasks. On the other hand, the task used a custom Android application, fixed product categories, repeated trials, and constrained product sets, and task difficulty was manipulated experimentally by varying the number of target products. Therefore, the paradigm is best understood as an ecologically oriented laboratory model of selected smartphone-interaction operations under cognitive load, rather than as a comprehensive assessment of everyday smartphone competence.

### 4.6. Limitations and Future Directions

Several limitations should be acknowledged. First, the sample sizes were modest, especially in Experiment 2 and in the active-group EEG–behavior correlation analyses. Although Experiment 1 provided short-term behavioral evidence, the EEG findings in Experiment 2 should be interpreted as exploratory and hypothesis-generating. Larger samples are needed to confirm the theta-band effects, test individual-difference moderators, and estimate the stability of EEG–behavior associations more precisely.

Experiment 1 and Experiment 2 also differed in timing and measurement structure. Experiment 1 used a different-day behavioral design, whereas Experiment 2 used a same-session reduced-trial EEG protocol in an independent sample. This difference does not represent a within-participant order confound across experiments, but it limits direct comparability between the two experiments and supports the interpretation of Experiment 2 as an EEG extension rather than a direct replication. Neither experiment used a crossover design. Although sham stimulation provided partial control for nonspecific effects, stimulus-set differences, task familiarization, fatigue, procedural learning, and session-related factors cannot be fully ruled out. Future studies should use counterbalanced crossover designs with independently validated equivalent stimulus sets.

Second, Experiment 2 was not a fully powered behavioral replication of Experiment 1. The number of trials was reduced to accommodate EEG recording and reduce participant burden. This design choice was appropriate for the EEG extension, but it likely reduced the reliability of efficiency-related behavioral measures such as completion time and difficulty–time slope. Future studies could use longer but tolerable EEG protocols, adaptive trial structures, or trial-level modeling to improve measurement precision while retaining ecological orientation and experimental control.

Third, EEG data quality varied across sessions and groups, as indicated by the Group × Time interaction in rejected-epoch proportions. Although this issue does not directly account for the behavioral target-selection-accuracy effect, it complicates the interpretation of pre-to-post EEG power changes. Future work should incorporate richer EEG quality metrics, preregistered artifact-control procedures, and sensitivity analyses that test whether oscillatory findings remain robust after controlling for data quality.

Fourth, the present EEG design cannot establish online entrainment. Because EEG was recorded before and after stimulation rather than during stimulation, the results speak to post-stimulation task-related oscillatory modulation. Future studies could combine stimulation-compatible EEG, individualized stimulation frequencies, phase-specific analyses, or concurrent neuroimaging approaches to test entrainment and network-level mechanisms more directly. Connectivity and cross-frequency coupling analyses may also be useful, given evidence that working memory depends on coordinated activity across frontoparietal networks and interactions between theta and higher-frequency rhythms [56,57].

Fifth, the present study used a single-session stimulation design. It remains unclear whether repeated HD-tACS sessions would produce larger, more durable, or more generalizable improvements. Prior work has shown that repeated neuromodulation can produce lasting improvements in memory in older adults [38], but whether such effects transfer to everyday smartphone tasks remains to be tested. Longitudinal studies are needed to determine whether repeated stimulation combined with smartphone training can produce durable improvements in digital functioning.

Sixth, the smartphone shopping task was designed to model cognitively demanding smartphone-task performance, not general smartphone proficiency. Smartphone use is heterogeneous, and different activities place different demands on memory, attention, motor control, language comprehension, risk evaluation, and social cognition. Future work should test whether the present findings generalize to other smartphone-based tasks and whether different cognitive mechanisms support different forms of digital behavior.

The smartphone-shopping paradigm should not be interpreted as a direct measure of general smartphone proficiency, digital autonomy, or broad digital inclusion. These broader outcomes depend on multiple individual-difference and contextual factors, including education, prior technological experience, interface familiarity, visual and motor abilities, self-efficacy, and technology anxiety. These variables were not comprehensively characterized in the present study and therefore could not be incorporated as predictors, covariates, or stratification factors. Participants were also relatively healthy older adults with daily smartphone experience, and the findings may not generalize to older adults with low digital literacy, cognitive impairment, sensory limitations, motor difficulties, or limited smartphone exposure.

The present task used a fixed-cardinality submission rule: participants were required to submit exactly the same number of products as the number of instructed targets. Accordingly, target selection accuracy captured both correct target retention and final distractor selection, because each retained distractor necessarily displaced one target product. Under this rule, the final distractor selection rate was equal to 1 − target selection accuracy, and complete-order success corresponded to a target selection accuracy of 100%. However, the available task logs did not retain the product-by-product interaction history needed to examine temporary distractor selections, deselections, cart-correction behavior, error-recovery strategies, or the temporal sequence of selection actions before final submission. Future task versions should log all selection, deselection, and correction actions with timestamps to allow process-level analyses of search behavior, error detection, and correction.

The stimulation montage was CP5-centered and scalp-based. Although this placement was theoretically motivated by parietal working-memory and technological-cognition literature, individualized MRI-based targeting and participant-specific electric-field modeling were not performed. Therefore, the results should not be interpreted as evidence of anatomically precise IPL stimulation.

Formal blinding efficacy could not be quantified because participants were queried only informally during debriefing, and forced-choice condition guesses, confidence ratings, and systematic adverse-sensation ratings by group were not collected. Although the sham procedure was designed to mimic the initial sensory experience of stimulation, future studies should incorporate formal blinding checks and standardized, condition-specific sensation and tolerability ratings. Future work should also assess prior smartphone proficiency, technology anxiety, and mobile self-efficacy to clarify which older adults may benefit most from HD-tACS and under what conditions.

## 5. Conclusions

The present study provides short-term behavioral evidence that CP5-centered parietal HD-tACS is associated with improved performance in a controlled smartphone-based shopping task in older adults. In Experiment 1, active stimulation reduced high-demand completion time, improved target selection accuracy, reduced the difficulty–time slope, and improved two-back working-memory performance, and trial-level sensitivity analyses supported the robustness of the main behavioral pattern. Experiment 2, which used a smaller reduced-trial EEG protocol, showed stimulation-related improvement in target selection accuracy and exploratory post-stimulation EEG associations between posterior/parietal theta-power changes and high-demand target selection accuracy improvement. These neural findings are exploratory and not definitive; they do not demonstrate online entrainment or mediation by theta activity. Overall, the findings suggest that parietal neuromodulation may influence selected working-memory and attentional-control components of cognitively demanding smartphone-task performance, providing a useful step toward integrating neuromodulation, cognitive aging, and human–technology interaction research.

## Figures and Tables

**Table 1:** Descriptive statistics for smartphone-task performance in Experiment 1.

Stimulation Group	Dependent Variable	Measurement Time	Low Difficulty *M* (*SD*)	Medium Difficulty *M* (*SD*)	High Difficulty *M* (*SD*)
Active HD-tACS	Completion time (s)	Pre-stimulation	39.62 (9.83)	53.56 (11.96)	67.40 (13.28)
		Post-stimulation	39.33 (8.45)	49.68 (10.38)	58.10 (12.24)
	Target selection accuracy (%)	Pre-stimulation	79.09 (16.54)	64.91 (23.66)	45.55 (13.22)
		Post-stimulation	83.42 (12.48)	72.04 (20.28)	58.05 (15.15)
	Difficulty–time slope	Pre-stimulation	Overall *M* (*SD*): 13.89 (7.12)		
		Post-stimulation	Overall *M* (*SD*): 9.39 (4.70)		
Sham	Completion time (s)	Pre-stimulation	40.17 (9.01)	51.73 (10.72)	66.41 (11.35)
		Post-stimulation	39.80 (8.18)	50.67 (10.32)	65.37 (10.32)
	Target selection accuracy (%)	Pre-stimulation	81.50 (13.72)	61.78 (14.91)	49.60 (13.58)
		Post-stimulation	82.26 (12.50)	63.02 (15.63)	51.50 (12.97)
	Difficulty–time slope	Pre-stimulation	Overall *M* (*SD*): 13.12 (6.12)		
		Post-stimulation	Overall *M* (*SD*): 12.79 (6.35)		

Note. Values are reported as *M* (*SD*). Target selection accuracy was defined as the proportion of instructed target products correctly retained in the submitted response. Because each trial required submission of exactly the same number of products as instructed targets, the final distractor selection rate was equal to 1 − target selection accuracy. Completion time is reported in seconds. Target selection accuracy is reported as a percentage. Lower completion time and lower difficulty–time slope indicate better performance, whereas higher target selection accuracy indicates better performance.

**Table 2:** Descriptive statistics for two-back working-memory performance in Experiment 1.

Stimulation Group	Dependent Variable	Measurement Time	*M* (*SD*)
Active HD-tACS	*RT* (s)	Pre-stimulation	1.30 (0.12)
		Post-stimulation	1.26 (0.09)
	Accuracy (%)	Pre-stimulation	64.38 (8.42)
		Post-stimulation	80.38 (4.68)
	*LISAS*	Pre-stimulation	1.48 (0.14)
		Post-stimulation	1.40 (0.10)
Sham	*RT* (s)	Pre-stimulation	1.36 (0.11)
		Post-stimulation	1.38 (0.13)
	Accuracy (%)	Pre-stimulation	66.63 (8.36)
		Post-stimulation	67.50 (8.74)
	*LISAS*	Pre-stimulation	1.52 (0.11)
		Post-stimulation	1.58 (0.15)

Note. Values are reported as *M* (*SD*). *RT* and *LISAS* are reported in seconds. Accuracy is reported as a percentage. Lower *RT* and lower *LISAS* indicate better performance, whereas higher accuracy indicates better performance.

**Table 3:** Descriptive statistics for smartphone-task performance in Experiment 2.

Stimulation Group	Dependent Variable	Measurement Time	Low Difficulty *M* (*SD*)	Medium Difficulty *M* (*SD*)	High Difficulty *M* (*SD*)
Active HD-tACS	Task completion time (s)	Pre-stimulation	40.53 (10.73)	49.38 (12.98)	57.55 (16.64)
		Post-stimulation	39.02 (10.58)	47.27 (12.82)	54.23 (14.77)
	Target selection accuracy (%)	Pre-stimulation	76.40 (9.90)	60.20 (10.20)	47.70 (11.80)
		Post-stimulation	79.30 (9.40)	65.30 (8.90)	56.00 (9.60)
	Difficulty–time slope	Pre-stimulation	8.51 (4.53)	—	—
		Post-stimulation	7.60 (4.01)	—	—
Sham	Task completion time (s)	Pre-stimulation	38.78 (5.84)	50.79 (10.21)	62.67 (11.69)
		Post-stimulation	38.34 (4.46)	49.06 (7.13)	61.31 (11.04)
	Target selection accuracy (%)	Pre-stimulation	78.70 (14.00)	62.10 (16.80)	49.80 (14.70)
		Post-stimulation	79.50 (13.30)	63.50 (17.80)	51.70 (13.00)
	Difficulty–time slope	Pre-stimulation	11.95 (6.08)	—	—
		Post-stimulation	11.49 (5.91)	—	—

Note. Values are reported as *M* (*SD*). Completion time is reported in seconds. Target selection accuracy is reported as a percentage. In each trial, participants were required to submit exactly the same number of products as instructed targets; therefore, the final distractor selection rate was equal to 1 − target selection accuracy. The difficulty–time slope indexes the increase in completion time as task difficulty increases. Lower completion time and lower difficulty–time slope indicate better performance, whereas higher target selection accuracy indicates better performance.

**Table 4:** Descriptive statistics for theta-band ROI power in Experiment 2.

Stimulation Group	Measurement Time	Frontal *M* (*SD*)	Central *M* (*SD*)	Temporal *M* (*SD*)	IPL *M* (*SD*)	PO *M* (*SD*)
Active HD-tACS	Pre-stimulation	3.47 (1.51)	2.47 (1.06)	1.24 (0.37)	1.60 (0.58)	2.17 (0.72)
	Post-stimulation	4.42 (2.71)	2.91 (1.55)	1.44 (0.56)	1.81 (0.70)	2.48 (0.89)
	Change	0.94 (1.60)	0.44 (0.63)	0.19 (0.25)	0.21 (0.22)	0.31 (0.32)
Sham	Pre-stimulation	3.71 (1.52)	2.34 (0.76)	1.24 (0.33)	1.49 (0.36)	1.88 (0.44)
	Post-stimulation	3.77 (1.66)	2.41 (0.76)	1.28 (0.32)	1.53 (0.39)	1.95 (0.46)
	Change	0.07 (0.97)	0.07 (0.36)	0.04 (0.15)	0.05 (0.13)	0.07 (0.17)

Note. Values are reported as *M* (*SD*). Theta power values represent mean PSD values extracted from task epochs. Change was calculated as post-stimulation minus pre-stimulation. PO = posterior parieto-occipital ROI.

**Table 5:** Hypothesis-guided correlations between high-difficulty target-selection-accuracy improvement and theta-power change in the active HD-tACS group.

ROI	*n*	*df*	*r*	Raw *p*	FDR-Adjusted *p*
Frontal	15	13	0.479	0.071	0.071
Central	15	13	0.491	0.063	0.071
Temporal	15	13	0.635	0.011	0.018 *
IPL	15	13	0.747	0.001	0.007 **
PO	15	13	0.663	0.007	0.018 *

Note. Correlations were conducted within the active HD-tACS group. High-difficulty target selection accuracy improvement was calculated as post-stimulation minus pre-stimulation. Theta-power change was calculated as post-stimulation minus pre-stimulation. FDR-adjusted *p*-values were calculated across the five hypothesis-guided ROI-level tests. Asterisks indicate significance based on FDR-adjusted *p*-values. PO = posterior parieto-occipital ROI. * *p* < 0.05, ** *p* < 0.01.

## Data Availability

The processed data supporting the findings of this study are openly available in OSF at https://osf.io/4jd3k/ (accessed on 1 June 2026).

## Supplementary Materials

The following supporting information can be downloaded at: https://www.mdpi.com/article/10.3390/brainsci16070678/s1, Table S1: Full exploratory ROI-level correlations between behavioral improvement and theta-power change in the active HD-tACS group; Table S2: Descriptive statistics for EEG ROI power across theta, alpha, beta, and gamma bands in Experiment 2; Table S3: Behavioral ANOVA summary for Experiments 1 and 2; Table S4: EEG power ANOVA summary for Experiment 2; Table S5: Baseline group comparisons and EEG rejected-epoch quality-control summary; Table S6: Follow-up and simple-effect summary for revised analyses; Table S7: Trial-level Gaussian GEE sensitivity analyses for Experiment 1 completion time; Table S8: Descriptive statistics for target hits, target omissions, target selection accuracy, derived final distractor selection rate, and complete-order success in Experiment 1; Table S9: Baseline-change correlations and ceiling/floor checks for smartphone-task outcomes; Table S10: Between-group differences in change scores with 95% confidence intervals; Table S11: EEG data-quality sensitivity analyses for theta-power changes and theta–behavior associations; Figure S1: Full exploratory ROI-level correlations between behavioral improvement and theta-power change in the active HD-tACS group; Figure S2: EEG rejected-epoch proportions by group and measurement time.
