# Supplementary material for: CP5-Centered Parietal HD-tACS Is Associated with Improved Performance in a Smartphone-Based Shopping Task in Older Adults: A Behavioral and EEG Investigation
Source: Brain Sci. 2026 Jun 27;16(7):678. doi: 10.3390/brainsci16070678 (PMC13406462; doi:10.3390/brainsci16070678)
Supplement: Supplementary file 1 [file brainsci-16-00678-s001.zip › brainsci-4382716-supplementary.pdf]

Supplementary Materials

CP5-Centered Parietal HD-tACS Is Associated with Improved Performance in a Smartphone-Based Shopping Task in Older Adults: A Behavioral and EEG Investigation

This supplementary document contains the full exploratory correlation matrix, EEG power descriptives, ANOVA summaries, baseline and data-quality checks, follow-up tests, trial-level sensitivity analyses, selection-component descriptives, change-score confidence intervals, and EEG data-quality sensitivity analyses that support the revised manuscript.

Table and figure numbering follows the order cited in the manuscript. All values are reported as *M* (*SD*) unless otherwise stated.

Supplementary Table S1. Full exploratory ROI-level correlations between behavioral improvement and theta-power change in the active HD-tACS group.

Behavioral improvement index	Theta ROI	*r*	raw *p*	FDR-adjusted *p*	FDR significant?
Low completion-time improvement	Frontal	−0.69	0.004**	0.075	No
	Central	−0.683	0.005**	0.075	No
	Temporal	−0.527	0.043*	0.217	No
	IPL	−0.445	0.096	0.261	No
	PO	−0.446	0.095	0.261	No
Medium completion-time improvement	Frontal	−0.256	0.357	0.473	No
	Central	−0.305	0.270	0.398	No
	Temporal	−0.476	0.073	0.252	No
	IPL	−0.335	0.222	0.385	No
	PO	−0.354	0.195	0.366	No
High completion-time improvement	Frontal	−0.239	0.391	0.488	No
	Central	−0.306	0.267	0.398	No
	Temporal	−0.297	0.283	0.398	No
	IPL	−0.245	0.379	0.487	No
	PO	−0.299	0.280	0.398	No
Mean completion-time improvement	Frontal	−0.498	0.059	0.252	No
	Central	−0.55	0.034*	0.216	No
	Temporal	−0.539	0.038*	0.216	No

Behavioral improvement index	Theta ROI	*r*	raw *p*	FDR-adjusted *p*	FDR significant?
Low target-selection-accuracy improvement	IPL	−0.43	0.110	0.261	No
	PO	−0.468	0.079	0.253	No
	Frontal	0.343	0.211	0.380	No
	Central	0.359	0.188	0.366	No
	Temporal	0.275	0.320	0.437	No
Medium target-selection-accuracy improvement	IPL	0.38	0.162	0.332	No
	PO	0.314	0.254	0.398	No
	Frontal	0.02	0.942	0.964	No
	Central	−0.055	0.845	0.884	No
	Temporal	−0.136	0.630	0.726	No
High target-selection-accuracy improvement	IPL	−0.068	0.809	0.867	No
	PO	−0.141	0.617	0.726	No
	Frontal	0.479	0.071	0.252	No
	Central	0.491	0.063	0.252	No
	Temporal	0.635	0.011*	0.099	No
Mean target-selection-accuracy improvement	IPL	0.747	0.001**	0.062	No
	PO	0.663	0.007**	0.080	No
	Frontal	0.43	0.110	0.261	No
	Central	0.403	0.136	0.292	No
	Temporal	0.403	0.136	0.292	No

Behavioral improvement index	Theta ROI	*r*	raw *p*	FDR-adjusted *p*	FDR significant?
	IPL	0.548	0.034*	0.216	No
	PO	0.433	0.107	0.261	No
Difficulty–time slope improvement	Frontal	0.313	0.256	0.398	No
	Central	0.215	0.443	0.538	No
	Temporal	0.083	0.770	0.860	No
	IPL	0.078	0.784	0.860	No
	PO	0.005	0.986	0.986	No

Note. Correlations were conducted within the active HD-tACS group ($n = 15$, $df = 13$). Completion-time and Difficulty–time-slope improvements were coded as pre-stimulation minus post-stimulation; target-selection-accuracy improvement and theta-power change were coded as post-stimulation minus pre-stimulation. Raw *p* values are marked with asterisks (* $p < 0.05$, ** $p < 0.01$). FDR-adjusted *p* values were calculated across the full 45-test exploratory matrix. No correlation survived FDR correction across this full matrix. The hypothesis-guided five-ROI correlation family reported in manuscript Table 5 was corrected separately.

Supplementary Table S2. Descriptive statistics for EEG ROI power across theta, alpha, beta, and gamma bands in Experiment 2.

Band	Stimulation group	ROI	Pre-stimulation *M* (*SD*)	Post-stimulation *M* (*SD*)	Change *M* (*SD*)	*n*
Theta	Active HD-tACS	Frontal	3.474 (1.512)	4.415 (2.714)	0.941 (1.601)	15
		Central	2.474 (1.063)	2.911 (1.548)	0.437 (0.629)	15
		Temporal	1.243 (0.368)	1.435 (0.564)	0.192 (0.251)	15
		IPL	1.601 (0.583)	1.811 (0.7)	0.21 (0.217)	15
		PO	2.171 (0.718)	2.476 (0.894)	0.305 (0.317)	15
	Sham	Frontal	3.707 (1.52)	3.773 (1.657)	0.066 (0.966)	13
		Central	2.343 (0.755)	2.409 (0.761)	0.067 (0.36)	13
		Temporal	1.237 (0.331)	1.278 (0.317)	0.041 (0.146)	13
		IPL	1.486 (0.355)	1.532 (0.388)	0.047 (0.131)	13
		PO	1.882 (0.435)	1.953 (0.455)	0.071 (0.173)	13
Alpha	Active HD-tACS	Frontal	1.555 (0.992)	1.765 (1.019)	0.21 (0.233)	15
		Central	1.39 (0.952)	1.525 (0.946)	0.134 (0.2)	15
		Temporal	0.897 (0.545)	0.994 (0.657)	0.097 (0.273)	15
		IPL	1.181 (0.489)	1.338 (0.553)	0.157 (0.216)	15
		PO	1.552 (0.472)	1.795 (0.579)	0.243 (0.295)	15
	Sham	Frontal	1.391 (0.682)	1.431 (0.735)	0.04 (0.209)	13
		Central	1.126 (0.572)	1.18 (0.618)	0.055 (0.157)	13

Band	Stimulation group	ROI	Pre-stimulation *M* (*SD*)	Post-stimulation *M* (*SD*)	Change *M* (*SD*)	*n*
		Temporal	0.68 (0.267)	0.718 (0.316)	0.038 (0.081)	13
		IPL	1.04 (0.539)	1.127 (0.633)	0.087 (0.155)	13
		PO	1.347 (0.632)	1.474 (0.79)	0.127 (0.237)	13
Beta	Active HD-tACS	Frontal	0.489 (0.286)	0.518 (0.187)	0.03 (0.309)	15
		Central	0.462 (0.3)	0.474 (0.187)	0.012 (0.324)	15
		Temporal	0.427 (0.327)	0.431 (0.222)	0.004 (0.284)	15
		IPL	0.551 (0.336)	0.594 (0.326)	0.043 (0.387)	15
		PO	0.707 (0.383)	0.793 (0.408)	0.086 (0.453)	15
	Sham	Frontal	0.438 (0.192)	0.447 (0.166)	0.009 (0.088)	13
		Central	0.383 (0.147)	0.405 (0.15)	0.022 (0.074)	13
		Temporal	0.321 (0.111)	0.334 (0.146)	0.013 (0.078)	13
		IPL	0.491 (0.272)	0.535 (0.336)	0.044 (0.109)	13
		PO	0.637 (0.324)	0.687 (0.395)	0.05 (0.127)	13
Gamma	Active HD-tACS	Frontal	0.193 (0.193)	0.195 (0.119)	0.001 (0.205)	15
		Central	0.201 (0.2)	0.2 (0.124)	−0.001 (0.213)	15
		Temporal	0.246 (0.221)	0.245 (0.152)	−0.002 (0.185)	15
		IPL	0.302 (0.243)	0.325 (0.224)	0.023 (0.263)	15
		PO	0.403 (0.298)	0.444 (0.264)	0.04 (0.322)	15

Band	Stimulation group	ROI	Pre-stimulation *M* (*SD*)	Post-stimulation *M* (*SD*)	Change *M* (*SD*)	*n*
	Sham	Frontal	0.148 (0.102)	0.162 (0.098)	0.014 (0.046)	13
		Central	0.146 (0.076)	0.165 (0.084)	0.019 (0.044)	13
		Temporal	0.167 (0.066)	0.175 (0.086)	0.007 (0.059)	13
		IPL	0.261 (0.141)	0.287 (0.182)	0.025 (0.068)	13
		PO	0.354 (0.175)	0.386 (0.221)	0.032 (0.084)	13

Note. Values represent mean PSD values extracted from task epochs. Change was calculated as post-stimulation minus pre-stimulation. PO = posterior parieto-occipital ROI.

Supplementary Table S3. Behavioral ANOVA summary for Experiments 1 and 2.

Module	Dependent variable	Effect	*df*	*F*	*p*	partial η_p^2
Experiment 1	Shopping completion time	Group	1, 38	0.165	0.687	0.004
		Time	1, 38	9.314	0.004**	0.197
		Group × Time	1, 38	4.447	0.042*	0.105
		Difficulty	2, 76	139.76	< 0.001***	0.786
		Group × Difficulty	2, 76	0.788	0.458	0.02
		Time × Difficulty	2, 76	6.212	0.003**	0.141
		Group × Time × Difficulty	2, 76	4.756	0.011*	0.111
	Shopping target selection accuracy	Group	1, 38	0.263	0.611	0.007
		Time	1, 38	47.334	< 0.001***	0.555
		Group × Time	1, 38	24.537	< 0.001***	0.392
		Difficulty	2, 76	138.259	< 0.001***	0.784
		Group × Difficulty	2, 76	1.779	0.176	0.045
		Time × Difficulty	2, 76	4.006	0.022*	0.095
		Group × Time × Difficulty	2, 76	2.298	0.107	0.057
	Difficulty–time slope	Group	1, 38	0.501	0.484	0.013
		Time	1, 38	19.039	< 0.001***	0.334
		Group × Time	1, 38	14.21	< 0.001***	0.272

Module	Dependent variable	Effect	*df*	*F*	*p*	partial η_p^2
	2-back *RT*	Group	1, 38	7.813	0.008**	0.171
		Time	1, 38	0.767	0.387	0.02
		Group × Time	1, 38	3.595	0.066	0.086
	2-back *AC*	Group	1, 38	5.561	0.024*	0.128
		Time	1, 38	78.932	< 0.001***	0.675
		Group × Time	1, 38	63.41	< 0.001***	0.625
	2-back *LISAS*	Group	1, 38	10.291	0.003**	0.213
		Time	1, 38	0.324	0.572	0.008
		Group × Time	1, 38	12.892	< 0.001***	0.253
Experiment 2	Shopping completion time	Group	1, 26	0.304	0.586	0.012
		Time	1, 26	9.104	0.006**	0.259
		Group × Time	1, 26	0.925	0.345	0.034
		Difficulty	2, 52	92.143	< 0.001***	0.78
		Group × Difficulty	2, 52	3.281	0.045*	0.112
		Time × Difficulty	2, 52	1.073	0.350	0.04
		Group × Time × Difficulty	2, 52	0.33	0.720	0.013
	Shopping target selection accuracy	Group	1, 26	0	0.991	0

Module	Dependent variable	Effect	*df*	*F*	*p*	partial η_p^2
		Time	1, 26	26.967	< 0.001***	0.509
		Group × Time	1, 26	9.149	0.006**	0.26
		Difficulty	2, 52	164.979	< 0.001***	0.864
		Group × Difficulty	2, 52	0.296	0.745	0.011
		Time × Difficulty	2, 52	2.721	0.075	0.095
		Group × Time × Difficulty	2, 52	1.106	0.339	0.041
	Difficulty–time slope	Group	1, 26	3.6	0.069	0.122
		Time	1, 26	6.432	0.018*	0.198
		Group × Time	1, 26	0.654	0.426	0.025

Note. This table reports the behavioral mixed-design ANOVA results used in the revised manuscript. Raw *p* values are marked with asterisks (* $p < 0.05$, ** $p < 0.01$, *** $p < 0.001$). Partial η_p^2 = partial eta squared.

Supplementary Table S4. EEG power ANOVA summary for Experiment 2.

Module	Dependent variable	Effect	*df*	*F*	*p*	partial η_p^2
Experiment 2 EEG	Theta power	Group	1, 26	0.565	0.459	0.021
		Time	1, 26	7.609	0.010*	0.226
		Group × Time	1, 26	3.875	0.060	0.13
		ROI	4, 104	63.595	< 0.001***	0.71
		Group × ROI	4, 104	0.246	0.912	0.009
		Time × ROI	4, 104	2.972	0.023*	0.103
		Group × Time × ROI	4, 104	2.354	0.059	0.083
	Alpha power	Group	1, 26	1.145	0.294	0.042
		Time	1, 26	12.036	0.002**	0.316
		Group × Time	1, 26	1.967	0.173	0.07
		ROI	4, 104	23.345	< 0.001***	0.473
		Group × ROI	4, 104	0.14	0.967	0.005
		Time × ROI	4, 104	3.667	0.008**	0.124
		Group × Time × ROI	4, 104	0.938	0.445	0.035
	Beta power	Group	1, 26	0.932	0.343	0.035
		Time	1, 26	0.407	0.529	0.015
		Group × Time	1, 26	0.006	0.941	0

Module	Dependent variable	Effect	*df*	*F*	*p*	partial η_p^2
		ROI	4, 104	23.854	< 0.001***	0.478
		Group × ROI	4, 104	0.12	0.975	0.005
		Time × ROI	4, 104	3.054	0.020*	0.105
		Group × Time × ROI	4, 104	0.493	0.741	0.019
	Gamma power	Group	1, 26	0.934	0.343	0.035
		Time	1, 26	0.22	0.643	0.008
		Group × Time	1, 26	0.011	0.916	0
		ROI	4, 104	41.246	< 0.001***	0.613
		Group × ROI	4, 104	0.249	0.910	0.009
		Time × ROI	4, 104	1.862	0.123	0.067
		Group × Time × ROI	4, 104	0.256	0.905	0.01

Note. This table reports the EEG power ANOVA results for theta, alpha, beta, and gamma bands. Raw *p* values are marked with asterisks (* $p < 0.05$, ** $p < 0.01$, *** $p < 0.001$). Partial η_p^2 = partial eta squared.

Supplementary Table S5. Baseline group comparisons and EEG rejected-epoch quality-control summary.

A. Baseline group comparisons

Section	Variable	*n active*	*n sham*	Active *M* (*SD*)	Sham *M* (*SD*)	*df*	*t*	*p*	Cohen's *d*
Experiment 1 smartphone task	Completion time - low difficulty (s)	20	20	39.622 (9.829)	40.17 (9.014)	38	−0.184	0.855	−0.058
	Completion time - medium difficulty (s)	20	20	53.562 (11.96)	51.725 (10.719)	38	0.512	0.612	0.162
	Completion time - high difficulty (s)	20	20	67.4 (13.277)	66.405 (11.346)	38	0.255	0.800	0.081
	Target selection accuracy - low difficulty	20	20	0.791 (0.165)	0.815 (0.137)	38	−0.503	0.618	−0.159
	Target selection accuracy - medium difficulty	20	20	0.649 (0.237)	0.617 (0.149)	38	0.5	0.620	0.158
	Target selection accuracy - high difficulty	20	20	0.456 (0.132)	0.496 (0.136)	38	−0.956	0.345	−0.302
	Difficulty–time slope	20	20	13.889 (7.124)	13.117 (6.117)	38	0.367	0.715	0.116
Experiment 1 2-back task	2-back *RT* (s)	20	20	1.301 (0.118)	1.363 (0.109)	38	−1.72	0.094	−0.544
	2-back accuracy	20	20	0.644 (0.084)	0.666 (0.084)	38	−0.848	0.402	−0.268
	2-back *LISAS*	20	20	1.482 (0.14)	1.524 (0.105)	38	−1.067	0.293	−0.337
Experiment 2 smartphone task	Completion time - low difficulty (s)	15	13	40.533 (10.73)	38.777 (5.835)	26	0.526	0.603	0.199
	Completion time - medium difficulty (s)	15	13	49.38 (12.977)	50.785 (10.208)	26	−0.315	0.756	−0.119
	Completion time - high difficulty (s)	15	13	57.553 (16.636)	62.669 (11.692)	26	−0.927	0.362	−0.351
	Target selection accuracy - low difficulty	15	13	0.764 (0.099)	0.787 (0.14)	26	−0.501	0.621	−0.19
	Target selection accuracy - medium difficulty	15	13	0.602 (0.102)	0.621 (0.168)	26	−0.376	0.710	−0.142

Section	Variable	*n active*	*n sham*	Active *M* (*SD*)	Sham *M* (*SD*)	*df*	*t*	*p*	Cohen's *d*
	Target selection accuracy - high difficulty	15	13	0.477 (0.118)	0.498 (0.147)	26	−0.422	0.677	−0.16
	Difficulty–time slope	15	13	8.51 (4.532)	11.946 (6.081)	26	−1.71	0.099	−0.648

Note. Baseline comparisons were conducted before testing stimulation effects. For target selection accuracy, values are proportions. No baseline group difference reached conventional significance.

B. Rejected-epoch proportion quality-control analysis

Effect	*df*	*F*	*p*	partial η_p^2
Group	1, 26	0.652	0.427	0.024
Time	1, 26	0.030	0.864	0.001
Group × Time	1, 26	5.109	0.032*	0.164

Note. The Group × Time interaction was recomputed from the retained/rejected epoch records used in the current revision.

C. Rejected-epoch descriptive statistics

Group	Time	*n*	Raw epochs, *M* (*SD*)	Retained epochs, *M* (*SD*)	Rejected epochs, *M* (*SD*)	Rejected proportion, *M* (*SD*)
Active HD-tACS	Pre-stimulation	15	150.1 (0.3)	108.1 (28.3)	42.0 (28.2)	0.280 (0.188)
Active HD-tACS	Post-stimulation	15	150.5 (1.8)	96.5 (34.6)	54.1 (35.2)	0.358 (0.232)
Sham	Pre-stimulation	13	152.5 (27.4)	104.7 (32.6)	47.8 (42.0)	0.306 (0.221)
Sham	Post-stimulation	13	150.0 (0.0)	116.3 (22.8)	33.7 (22.8)	0.225 (0.152)

Note. Rejected proportion was calculated as rejected epochs divided by raw epochs for each participant and session.

Supplementary Table S6. Follow-up and simple-effect summary for revised analyses.

Module	Group	DV/Band	Difficulty/ROI	Pre *M* (*SD*)	Post *M* (*SD*)	*df*	*t*	*p*	Cohen's d_z
Exp. 1 shopping completion time	Active HD-tACS	*Completion time*	3	39.622 (9.829)	39.33 (8.448)	19	−0.219	0.829	−0.049
			4	53.562 (11.96)	49.68 (10.381)	19	−1.839	0.082	−0.411
			5	67.4 (13.277)	58.102 (12.239)	19	−4.671	< 0.001***	−1.044
			mean	53.528 (9.542)	49.038 (9.536)	19	−3.429	0.003**	−0.767
	Sham		3	40.17 (9.014)	39.795 (8.181)	19	−0.445	0.661	−0.1
			4	51.725 (10.719)	50.67 (10.317)	19	−0.504	0.620	−0.113
			5	66.405 (11.346)	65.373 (10.321)	19	−0.89	0.384	−0.199
			mean	52.767 (8.492)	51.946 (7.426)	19	−0.716	0.483	−0.16
Exp. 1 shopping target selection accuracy	Active HD-tACS	Target selection accuracy	3	0.791 (0.165)	0.834 (0.125)	19	2.866	0.010**	0.641
			4	0.649 (0.237)	0.72 (0.203)	19	2.926	0.009**	0.654
			5	0.456 (0.132)	0.581 (0.152)	19	6.538	< 0.001***	1.462
			mean	0.632 (0.16)	0.712 (0.144)	19	7.413	< 0.001***	1.658
	Sham		3	0.815 (0.137)	0.823 (0.125)	19	0.709	0.487	0.159
			4	0.617 (0.149)	0.63 (0.156)	19	0.915	0.372	0.205
			5	0.496 (0.136)	0.515 (0.13)	19	1.431	0.169	0.32
			mean	0.643 (0.128)	0.656 (0.121)	19	1.599	0.126	0.358

Module	Group	DV/Band	Difficulty/ROI	Pre *M* (*SD*)	Post *M* (*SD*)	*df*	*t*	*p*	Cohen's d_z
Exp. 1 difficulty–time slope	Active HD-tACS	Slope	-	13.889 (7.124)	9.386 (4.698)	19	−4.335	< 0.001***	−0.969
	Sham		-	13.117 (6.117)	12.789 (6.349)	19	−0.857	0.402	−0.192
Exp. 1 2-back task	Active HD-tACS	*RT*	-	1.301 (0.118)	1.26 (0.092)	19	−2.166	0.043*	−0.484
	Sham		-	1.363 (0.109)	1.378 (0.126)	19	0.664	0.515	0.148
	Active HD-tACS	*AC*	-	0.644 (0.084)	0.804 (0.047)	19	9.66	< 0.001***	2.16
	Sham		-	0.666 (0.084)	0.675 (0.087)	19	0.941	0.358	0.21
	Active HD-tACS	*LISAS*	-	1.482 (0.14)	1.402 (0.095)	19	−3.787	0.001**	−0.847
	Sham		-	1.524 (0.105)	1.581 (0.149)	19	1.808	0.087	0.404
Exp. 2 shopping completion time	Active HD-tACS	*Completion time*	3	40.533 (10.73)	39.02 (10.581)	14	−2.136	0.051	−0.551
			4	49.38 (12.977)	47.267 (12.815)	14	−3.643	0.003**	−0.941
			5	57.553 (16.636)	54.227 (14.765)	14	−3.18	0.007**	−0.821
			mean	49.156 (13.124)	46.838 (12.353)	14	−3.899	0.002**	−1.007
	Sham		3	38.777 (5.835)	38.335 (4.455)	12	−0.429	0.676	−0.119
			4	50.785 (10.208)	49.062 (7.132)	12	−0.829	0.423	−0.23
			5	62.669 (11.692)	61.308 (11.037)	12	−1.186	0.259	−0.329
			mean	50.744 (7.608)	49.568 (5.999)	12	−1.091	0.297	−0.302

Module	Group	DV/Band	Difficulty/ROI	Pre M (SD)	Post M (SD)	df	t	p	Cohen's d_z
Exp. 2 shopping target selection accuracy	Active HD-tACS	Selection accuracy	3	0.764 (0.099)	0.793 (0.094)	14	2.694	0.017*	0.696
			4	0.602 (0.102)	0.653 (0.089)	14	3.969	0.001**	1.025
			5	0.477 (0.118)	0.56 (0.096)	14	6.122	< 0.001***	1.581
			mean	0.614 (0.099)	0.669 (0.084)	14	6.741	< 0.001***	1.74
	Sham		3	0.787 (0.14)	0.795 (0.133)	12	0.433	0.673	0.12
			4	0.621 (0.168)	0.635 (0.178)	12	0.675	0.512	0.187
			5	0.498 (0.147)	0.517 (0.13)	12	1.085	0.299	0.301
			mean	0.636 (0.143)	0.649 (0.132)	12	1.169	0.265	0.324
Exp. 2 difficulty–time slope	Active HD-tACS	Slope	-	8.51 (4.532)	7.603 (4.011)	14	−2.375	0.032*	−0.613
	Sham		-	11.946 (6.081)	11.487 (5.905)	12	−1.155	0.271	−0.32
Exp. 2 EEG ROI mean	Active HD-tACS	Theta	mean	2.193 (0.794)	2.61 (1.231)	14	2.783	0.015*	0.719
	Sham		mean	2.131 (0.635)	2.189 (0.671)	12	0.643	0.533	0.178
	Active HD-tACS	Alpha	mean	1.315 (0.659)	1.483 (0.693)	14	3.135	0.007**	0.809
	Sham		mean	1.117 (0.494)	1.186 (0.586)	12	1.58	0.140	0.438
	Active HD-tACS	Beta	mean	0.527 (0.316)	0.562 (0.242)	14	0.391	0.702	0.101
	Sham		mean	0.454 (0.178)	0.482 (0.219)	12	1.122	0.284	0.311

Module	Group	DV/Band	Difficulty/ROI	Pre *M* (*SD*)	Post *M* (*SD*)	*df*	*t*	*p*	Cohen's d_z
Exp. 2 EEG ROI-specific	Active HD-tACS	Gamma	mean	0.269 (0.226)	0.282 (0.166)	14	0.203	0.842	0.052
	Sham		mean	0.215 (0.096)	0.235 (0.122)	12	1.341	0.205	0.372
	Active HD-tACS	Theta	Frontal	3.474 (1.512)	4.415 (2.714)	14	2.276	0.039*	0.588
			Central	2.474 (1.063)	2.911 (1.548)	14	2.691	0.018*	0.695
			Temporal	1.243 (0.368)	1.435 (0.564)	14	2.957	0.010*	0.763
			IPL	1.601 (0.583)	1.811 (0.7)	14	3.762	0.002**	0.971
			PO	2.171 (0.718)	2.476 (0.894)	14	3.729	0.002**	0.963
	Sham		Frontal	3.707 (1.52)	3.773 (1.657)	12	0.248	0.808	0.069
			Central	2.343 (0.755)	2.409 (0.761)	12	0.668	0.517	0.185
			Temporal	1.237 (0.331)	1.278 (0.317)	12	1.011	0.332	0.28
			IPL	1.486 (0.355)	1.532 (0.388)	12	1.291	0.221	0.358
			PO	1.882 (0.435)	1.953 (0.455)	12	1.487	0.163	0.412
	Active HD-tACS	Alpha	Frontal	1.555 (0.992)	1.765 (1.019)	14	3.488	0.004**	0.901
			Central	1.39 (0.952)	1.525 (0.946)	14	2.608	0.021*	0.673
			Temporal	0.897 (0.545)	0.994 (0.657)	14	1.371	0.192	0.354
			IPL	1.181 (0.489)	1.338 (0.553)	14	2.806	0.014*	0.724
			PO	1.552 (0.472)	1.795 (0.579)	14	3.194	0.006**	0.825

Module	Group	DV/Band	Difficulty/ROI	Pre *M* (*SD*)	Post *M* (*SD*)	*df*	*t*	*p*	Cohen's d_z
	Sham		Frontal	1.391 (0.682)	1.431 (0.735)	12	0.684	0.507	0.19
			Central	1.126 (0.572)	1.18 (0.618)	12	1.256	0.233	0.348
			Temporal	0.68 (0.267)	0.718 (0.316)	12	1.679	0.119	0.466
			IPL	1.04 (0.539)	1.127 (0.633)	12	2.017	0.067	0.559
			PO	1.347 (0.632)	1.474 (0.79)	12	1.929	0.078	0.535
	Active HD-tACS	Beta	Frontal	0.489 (0.286)	0.518 (0.187)	14	0.371	0.716	0.096
			Central	0.462 (0.3)	0.474 (0.187)	14	0.145	0.887	0.037
			Temporal	0.427 (0.327)	0.431 (0.222)	14	0.055	0.957	0.014
			IPL	0.551 (0.336)	0.594 (0.326)	14	0.434	0.671	0.112
			PO	0.707 (0.383)	0.793 (0.408)	14	0.733	0.475	0.189
	Sham		Frontal	0.438 (0.192)	0.447 (0.166)	12	0.384	0.708	0.107
			Central	0.383 (0.147)	0.405 (0.15)	12	1.048	0.315	0.291
			Temporal	0.321 (0.111)	0.334 (0.146)	12	0.606	0.556	0.168
			IPL	0.491 (0.272)	0.535 (0.336)	12	1.451	0.173	0.402
			PO	0.637 (0.324)	0.687 (0.395)	12	1.418	0.182	0.393
	Active HD-tACS	Gamma	Frontal	0.193 (0.193)	0.195 (0.119)	14	0.027	0.979	0.007
			Central	0.201 (0.2)	0.2 (0.124)	14	−0.019	0.985	−0.005

Module	Group	DV/Band	Difficulty/ROI	Pre *M* (*SD*)	Post *M* (*SD*)	*df*	*t*	*p*	Cohen's d_z
			Temporal	0.246 (0.221)	0.245 (0.152)	14	−0.038	0.970	−0.01
			IPL	0.302 (0.243)	0.325 (0.224)	14	0.335	0.742	0.087
			PO	0.403 (0.298)	0.444 (0.264)	14	0.485	0.635	0.125
	Sham		Frontal	0.148 (0.102)	0.162 (0.098)	12	1.111	0.288	0.308
			Central	0.146 (0.076)	0.165 (0.084)	12	1.526	0.153	0.423
			Temporal	0.167 (0.066)	0.175 (0.086)	12	0.439	0.669	0.122
			IPL	0.261 (0.141)	0.287 (0.182)	12	1.338	0.206	0.371
			PO	0.354 (0.175)	0.386 (0.221)	12	1.379	0.193	0.382

Note. This table summarizes within-group pre–post follow-up tests and related simple effects used for figure annotations and text reporting. Values are reported as *M* (*SD*). Raw *p* values are marked with asterisks (* $p < 0.05$, ** $p < 0.01$, *** $p < 0.001$). For follow-up comparisons involving multiple task-difficulty levels, Bonferroni-adjusted *p* values are reported in the manuscript text. d_z = within-subject standardized mean difference.

Supplementary Table S7. Trial-level Gaussian GEE sensitivity analyses for Experiment 1 completion time.

Model	Wald test	*df*	Wald χ^2	*p*
All difficulties	Group	1	0.040	0.841
	Time	1	0.047	0.829
	Difficulty	2	96.489	< 0.001***
	Group × Time	1	0.007	0.936
	Group × Difficulty	2	4.475	0.107
	Time × Difficulty	2	16.894	< 0.001***
	Group × Time × Difficulty	2	13.387	0.001**
High difficulty only	Group	1	4.676	0.031*
	Time	1	23.242	< 0.001***
	Group × Time	1	15.186	< 0.001***

Note. The all-difficulties model included 4,800 trials nested within 40 participants and used log-transformed completion time with exchangeable within-participant correlation. The high-difficulty model included 1,600 trials. Tests are robust Wald χ^2 tests from Gaussian generalized estimating equations.

Supplementary Table S8. Descriptive statistics for target hits, target omissions, target selection accuracy, derived final distractor selection rate, and complete-order success in Experiment 1.

Group	Time	Difficulty	*n*	Target hits, *M* (*SD*)	Target omissions, *M* (*SD*)	Target selection accuracy (%), *M* (*SD*)	Final distractor selection rate (%), *M* (*SD*)	Complete-order success (%), *M* (*SD*)
Active HD-tACS	Pre-stimulation	Low (3 targets)	20	47.45 (9.92)	12.55 (9.92)	79.1 (16.5)	20.9 (16.5)	57.0 (29.5)
Active HD-tACS	Pre-stimulation	Medium (4 targets)	20	51.9 (18.93)	28.1 (18.93)	64.9 (23.7)	35.1 (23.7)	36.5 (30.7)
Active HD-tACS	Pre-stimulation	High (5 targets)	20	45.55 (13.22)	54.45 (13.22)	45.6 (13.2)	54.4 (13.2)	8.8 (8.6)
Active HD-tACS	Post-stimulation	Low (3 targets)	20	50.05 (7.49)	9.95 (7.49)	83.4 (12.5)	16.6 (12.5)	64.2 (24.0)
Active HD-tACS	Post-stimulation	Medium (4 targets)	20	57.6 (16.23)	22.4 (16.23)	72.0 (20.3)	28.0 (20.3)	42.8 (29.2)
Active HD-tACS	Post-stimulation	High (5 targets)	20	58.05 (15.15)	41.95 (15.15)	58.1 (15.2)	42.0 (15.2)	20.0 (15.4)
Sham	Pre-stimulation	Low (3 targets)	20	48.9 (8.23)	11.1 (8.23)	81.5 (13.7)	18.5 (13.7)	59.5 (24.8)
Sham	Pre-stimulation	Medium (4 targets)	20	49.4 (11.93)	30.6 (11.93)	61.7 (14.9)	38.2 (14.9)	25.8 (19.8)
Sham	Pre-stimulation	High (5 targets)	20	49.6 (13.58)	50.4 (13.58)	49.6 (13.6)	50.4 (13.6)	12.3 (12.3)
Sham	Post-stimulation	Low (3 targets)	20	49.35 (7.49)	10.65 (7.49)	82.2 (12.5)	17.8 (12.5)	61.3 (25.1)
Sham	Post-stimulation	Medium (4 targets)	20	50.4 (12.49)	29.6 (12.49)	63.0 (15.6)	37.0 (15.6)	26.5 (22.2)
Sham	Post-stimulation	High (5 targets)	20	51.5 (12.97)	48.5 (12.97)	51.5 (13.0)	48.5 (13.0)	12.5 (10.7)

Note. Each difficulty condition contained 20 trials in Experiment 1. Target hits and omissions are total counts per participant across the 20 trials in that condition. Participants were required to submit exactly the same number of products as the number of instructed targets. Therefore, the final distractor selection rate was equal to 1 − target selection accuracy and is reported as a derived descriptive index rather than an independent outcome. Complete-order success is the proportion of trials in which all instructed targets were correctly retained in the submitted response.

Supplementary Table S9. Baseline-change correlations and ceiling/floor checks for smartphone-task outcomes.

A. Correlations between baseline performance and pre–post change

Experiment	Outcome	*n*	*r [95% CI]*	Raw *p*	FDR-adjusted *p*
Exp. 1	Completion time - low difficulty	40	0.475 [0.192, 0.685]	0.002	0.003
Exp. 1	Target selection accuracy - low difficulty	40	−0.610 [−0.774, −0.368]	< 0.001	< 0.001
Exp. 1	Completion time - medium difficulty	40	0.521 [0.250, 0.716]	< 0.001	0.002
Exp. 1	Target selection accuracy - medium difficulty	40	−0.356 [−0.601, −0.050]	0.024	0.028
Exp. 1	Completion time - high difficulty	40	0.392 [0.092, 0.627]	0.012	0.017
Exp. 1	Target selection accuracy - high difficulty	40	−0.232 [−0.507, 0.086]	0.150	0.150
Exp. 1	Difficulty–time slope	40	0.492 [0.213, 0.697]	0.001	0.003
Exp. 2	Completion time - low difficulty	28	0.339 [−0.039, 0.632]	0.077	0.108
Exp. 2	Target selection accuracy - low difficulty	28	−0.353 [−0.642, 0.023]	0.065	0.108
Exp. 2	Completion time - medium difficulty	28	0.437 [0.076, 0.696]	0.020	0.070
Exp. 2	Target selection accuracy - medium difficulty	28	−0.224 [−0.551, 0.163]	0.252	0.252
Exp. 2	Completion time - high difficulty	28	0.399 [0.031, 0.672]	0.035	0.082
Exp. 2	Target selection accuracy - high difficulty	28	−0.499 [−0.735, −0.154]	0.007	0.049
Exp. 2	Difficulty–time slope	28	0.283 [−0.101, 0.593]	0.145	0.169

Note. Completion-time and Difficulty–time-slope improvements were coded as pre-stimulation minus post-stimulation. Target-selection-accuracy improvement was coded as post-stimulation minus pre-stimulation. FDR adjustment was performed separately within each experiment across the listed correlations.

B. Baseline ceiling and floor checks for target selection accuracy

Experiment	Group	Difficulty	*n*	*M (SD), %*	Min, %	Max, %	Ceiling ≥ 95%, *n*	Floor ≤ 5%, *n*
Exp. 1	Active HD-tACS	Low	20	79.1 (16.5)	38.3	100.0	5	0
Exp. 1	Sham	Low	20	81.5 (13.7)	43.3	98.3	3	0
Exp. 1	All participants	Low	40	80.3 (15.0)	38.3	100.0	8	0
Exp. 1	Active HD-tACS	Medium	20	64.9 (23.7)	25.0	97.5	1	0
Exp. 1	Sham	Medium	20	61.7 (14.9)	36.2	87.5	0	0

Experiment	Group	Difficulty	*n*	*M (SD), %*	Min, %	Max, %	Ceiling ≥ 95%, *n*	Floor ≤ 5%, *n*
Exp. 1	All participants	Medium	40	63.3 (19.6)	25.0	97.5	1	0
Exp. 1	Active HD-tACS	High	20	45.6 (13.2)	21.0	69.0	0	0
Exp. 1	Sham	High	20	49.6 (13.6)	24.0	75.0	0	0
Exp. 1	All participants	High	40	47.6 (13.4)	21.0	75.0	0	0
Exp. 2	Active HD-tACS	Low	15	76.4 (9.9)	53.3	90.0	0	0
Exp. 2	Sham	Low	13	78.7 (14.0)	43.3	96.7	1	0
Exp. 2	All participants	Low	28	77.5 (11.8)	43.3	96.7	1	0
Exp. 2	Active HD-tACS	Medium	15	60.2 (10.2)	40.0	80.0	0	0
Exp. 2	Sham	Medium	13	62.1 (16.8)	37.5	87.5	0	0
Exp. 2	All participants	Medium	28	61.1 (13.5)	37.5	87.5	0	0
Exp. 2	Active HD-tACS	High	15	47.7 (11.8)	26.0	72.0	0	0
Exp. 2	Sham	High	13	49.8 (14.7)	24.0	74.0	0	0
Exp. 2	All participants	High	28	48.7 (13.0)	24.0	74.0	0	0

Note. Ceiling and floor counts are based on each participant's baseline target selection accuracy at each difficulty level. The high-difficulty condition showed no baseline ceiling cases in either experiment.

Supplementary Table S10. Between-group differences in change scores with 95% confidence intervals.

Experiment	Outcome	Change coding	Active *M* (*SD*)	Sham *M* (*SD*)	Active − sham	95% CI	*t(df)*	*p*
Exp. 1	Completion time - low difficulty improvement	pre − post	0.293 (5.977)	0.375 (3.764)	−0.082	[−3.300, 3.135]	−0.052 (32.0)	0.959
Exp. 1	Target selection accuracy - low difficulty improvement	post − pre	0.043 (0.068)	0.008 (0.047)	0.036	[−0.002, 0.073]	1.942 (34.0)	0.061
Exp. 1	Completion time - medium difficulty improvement	pre − post	3.882 (9.443)	1.055 (9.353)	2.828	[−3.189, 8.844]	0.951 (38.0)	0.347
Exp. 1	Target selection accuracy - medium difficulty improvement	post − pre	0.071 (0.109)	0.012 (0.061)	0.059	[0.002, 0.116]	2.104 (29.9)	0.044
Exp. 1	Completion time - high difficulty improvement	pre − post	9.297 (8.903)	1.033 (5.187)	8.265	[3.563, 12.967]	3.587 (30.6)	0.001
Exp. 1	Target selection accuracy - high difficulty improvement	post − pre	0.125 (0.086)	0.019 (0.059)	0.106	[0.059, 0.153]	4.554 (33.9)	< 0.001
Exp. 1	Difficulty–time slope improvement	pre − post	4.503 (4.645)	0.329 (1.715)	4.174	[1.889, 6.459]	3.770 (24.1)	< 0.001
Exp. 2	Completion time - low difficulty improvement	pre − post	1.513 (2.744)	0.442 (3.721)	1.071	[−1.526, 3.668]	0.856 (21.8)	0.402
Exp. 2	Target selection accuracy - low difficulty improvement	post − pre	0.029 (0.042)	0.008 (0.064)	0.021	[−0.022, 0.064]	1.022 (20.1)	0.319
Exp. 2	Completion time - medium difficulty improvement	pre − post	2.113 (2.247)	1.723 (7.491)	0.390	[−4.240, 5.021]	0.181 (13.9)	0.859
Exp. 2	Target selection accuracy - medium difficulty improvement	post − pre	0.052 (0.050)	0.013 (0.072)	0.038	[−0.011, 0.088]	1.604 (21.1)	0.123
Exp. 2	Completion time - high difficulty improvement	pre − post	3.327 (4.052)	1.362 (4.140)	1.965	[−1.232, 5.163]	1.265 (25.3)	0.217
Exp. 2	Target selection accuracy - high difficulty improvement	post − pre	0.083 (0.052)	0.018 (0.061)	0.064	[0.019, 0.109]	2.956 (23.8)	0.007
Exp. 2	Difficulty–time slope improvement	pre − post	0.907 (1.479)	0.460 (1.435)	0.447	[−0.687, 1.582]	0.811 (25.6)	0.425

Note. For completion time and Difficulty–time slope, positive change reflects pre-stimulation minus post-stimulation. For target selection accuracy, positive change reflects post-stimulation minus pre-stimulation. Target-selection-accuracy values and their confidence intervals are expressed as proportions in this table.

Supplementary Table S11. EEG data-quality sensitivity analyses for theta-power changes and theta–behavior associations.

A. ANCOVA models for theta-power change adjusted for baseline theta power and change in rejected-epoch proportion

ROI	*n*	Active–sham β	95% CI	*p (group)*	FDR-adjusted *p*	Change in rejected-epoch proportion, β	*p (change in rejected-epoch proportion)*	Adjusted R^2
Mean	28	0.138	[−0.200, 0.475]	0.409	—	1.263	0.006	0.396
Frontal	28	0.234	[-0.656, 1.124]	0.592	0.592	4.591	< 0.001	0.444
Central	28	0.114	[−0.242, 0.47]	0.515	0.592	1.382	0.004	0.427
Temporal	28	0.105	[−0.064, 0.275]	0.213	0.354	0.277	0.197	0.187
IPL	28	0.134	[−0.019, 0.288]	0.082	0.342	0.095	0.617	0.184
PO	28	0.168	[−0.057, 0.394]	0.137	0.342	0.176	0.519	0.171

Note. The mean-ROI model is shown descriptively and was not included in the five-ROI FDR family. FDR-adjusted *p* values were calculated across the five ROI-specific group effects. No group-level theta-power change remained statistically reliable after adjustment for data-quality variation.

B. Active-group sensitivity analyses for correlations between theta-power change and high-difficulty target-selection-accuracy improvement

ROI	Raw *r* (FDR *p*)	Partial *r* controlling change in rejected-epoch proportion (FDR *p*)	Partial *r* controlling change in rejected-epoch proportion and baseline theta (FDR *p*)	*r after excluding high-artifact participants (FDR p)*
Frontal	0.479 (0.071)	0.681 (0.005)	0.723 (0.002)	0.345 (0.363)
Central	0.491 (0.071)	0.703 (0.004)	0.735 (0.002)	0.353 (0.363)
Temporal	0.635 (0.018)	0.785 (0.001)	0.785 (0.001)	0.532 (0.242)
IPL	0.747 (0.007)	0.808 (0.001)	0.809 (0.001)	0.639 (0.242)
PO	0.663 (0.018)	0.709 (0.004)	0.720 (0.002)	0.527 (0.242)

Note. All correlation families were corrected across the five ROIs. High-artifact participants were defined as those with more than 50% rejected epochs in either the pre- or post-stimulation EEG session; exclusion reduced the active group from $n = 15$ to $n = 9$. Raw and adjusted analyses use the active HD-tACS group only.

Supplementary Figure S1. Full exploratory ROI-level correlations between behavioral improvement and theta-power change in the active HD-tACS group.

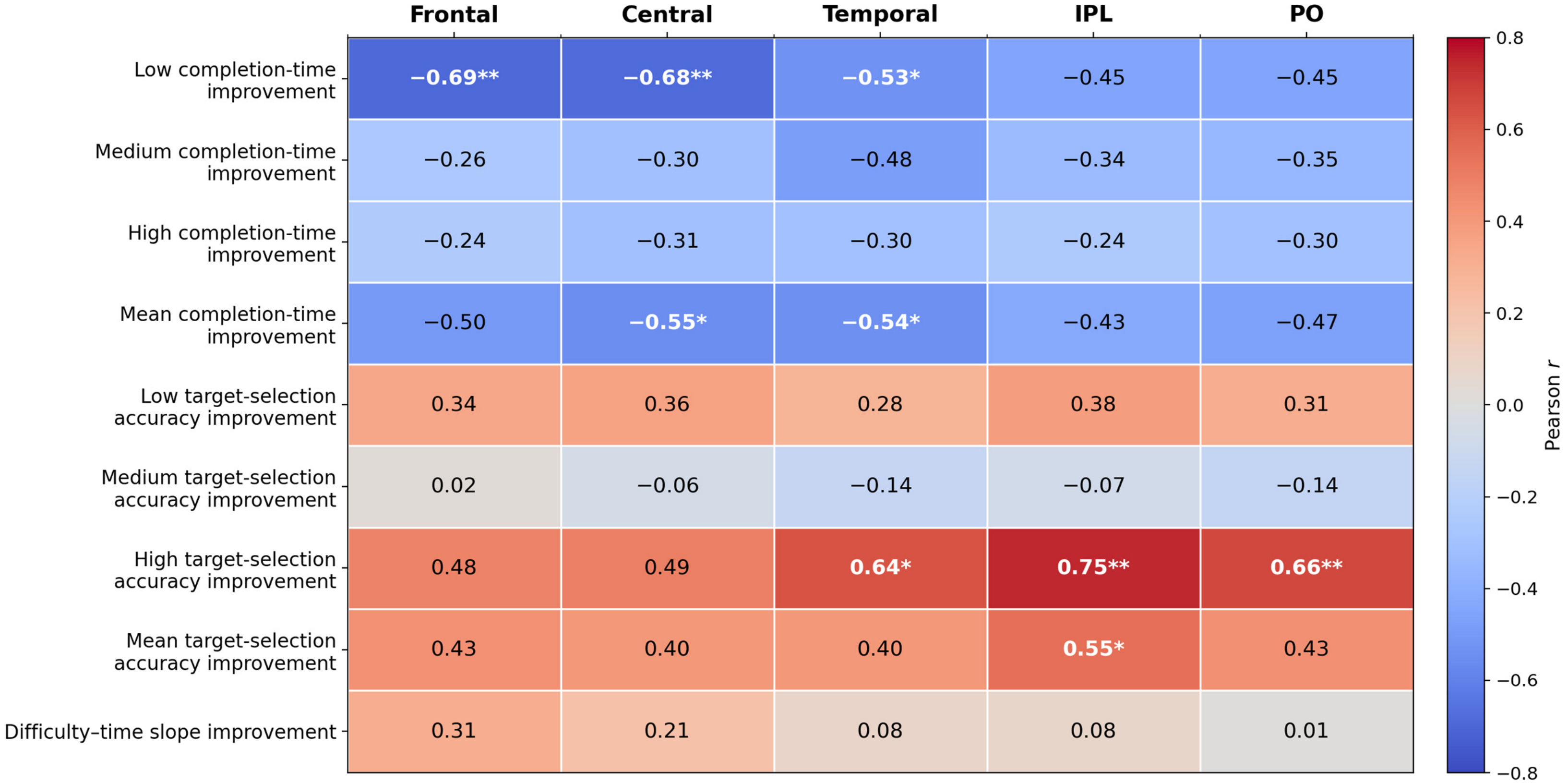

Note. Color indicates Pearson r. Asterisks indicate raw p values (* $p < 0.05$, ** $p < 0.01$). No correlation survived FDR correction across the full 45-test exploratory matrix. Exact raw p and FDR-adjusted p values are reported in Supplementary Table S1. $n = 15$, $df = 13$.

Supplementary Figure S2. EEG rejected-epoch proportions by group and measurement time.

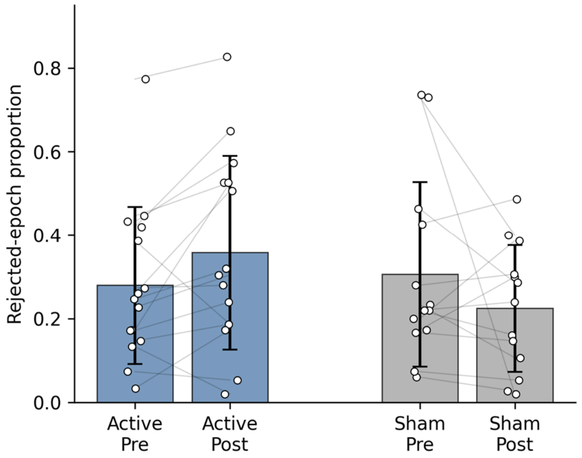

Note. Error bars show *SD*. Individual points and thin lines show participant-level data. The Group × Time interaction was $F(1, 26) = 5.109$, $p = 0.032$, $\eta_p^2 = 0.164$. The pattern is displayed to document data-quality variation and should be interpreted in conjunction with the sensitivity analyses in Supplementary Table S11.
